# Exploring personal aptitudes and personality traits that, together with social determinants, shape health behaviors and conduct: a thematic analysis based on the Capability, Opportunity, Motivation and Behavior (COM-B) change system

**DOI:** 10.3389/fpubh.2024.1387528

**Published:** 2024-06-05

**Authors:** Yudy Young-Silva, Anna Berenguera, Dolors Juvinyà-Canal, Ruth Martí-Lluch, Paula Arroyo-Uriarte, Olaya Tamayo-Morales, Irene Marcilla-Toribio, Usue Elizondo-Alzola, Fátima Méndez-López, Xènia Chela-Àlvarez, Emma Motrico

**Affiliations:** ^1^Unitat de Suport a la Recerca Girona, Fundació Institut Universitari per a la Recerca a l'Atenció Primària de Salut Jordi Goli Gurina (IDIAPJGol), Barcelona, Spain; ^2^Escola de doctorat, Universitat de Girona, Girona, España; ^3^Fundació Institut Universitari per a la Recerca a l'Atenció Primària de Salut Jordi Goli Gurina (IDIAPJGol), Barcelona, Spain; ^4^Department d’Infermeria, Universitat de Girona, Girona, Spain; ^5^Departament de Salut Pública. Universitat Autònoma de Barcelona, Bellaterra, España; ^6^Country Network on Chronicity, Primary Care, and Health Prevention and Promotion (RICAPPS), Bellaterra, Spain; ^7^Grup de Recerca Salut i Atenció sanitària Universitat de Girona, Girona, Spain; ^8^Càtedra de Promoció de la Salut Universitat de Girona, Girona, Spain; ^9^Vascular Health Research Group of Girona, Institut Universitari per a la Recerca a l’Atenció Primària Jordi Gol i Gurina (IDIAPJGol), Girona, Spain; ^10^Parc Hospitalari Martí Julià, Institut d'Investigació Biomèdica de Girona (IDIBGI), Salt, Spain; ^11^Research Group, Institut de Recerca Sant Joan de Déu, Esplugues de Llobregat, Spain; ^12^Unidad de Investigación en Atención Primaria de Salamanca (APISAL), Salamanca, Spain; ^13^Instituto de Investigación Biomédica de Salamanca (IBSAL), Salamanca, Spain; ^14^Centro de Estudios Sociosanitarios, Universidad de Castilla-La Mancha, Edificio Melchor Cano, Campus de Cuenca s/n, Cuenca, Spain; ^15^Grupo de Investigación Health, Gender, and Social Determinants, Universidad de Castilla-La Mancha, Edificio Melchor Cano, Campus de Cuenca s/n, Cuenca, Spain; ^16^Grupo de Investigación en Ciencias de la Diseminación e Implementación en Servicios Sanitarios, Instituto Investigación de Biocruces, Barakaldo, Spain; ^17^Osakidetza Basque Health Service, Debagoiena-Integrated Health Care Organization, Pharmacy Service (Primary Care), Arrasate, Gipuzkoa, Spain; ^18^Aragonese Primary Care Research Group (GAIAP), Institute for Health Research Aragón (IIS Aragón), Zaragoza, Spain; ^19^Primary Care Research Unit of Mallorca (IB-Salut), Balearic Health Service, Palma de Mallorca, Spain; ^20^Research Group in Primary Care and Promotion-Balearic Islands Community (GRAPP-caIB), Health Research Institute of the Balearic Islands (IdISBa), Palma de Mallorca, Spain; ^21^Department of Developmental and Educational Psychology, Institute of Biomedicine of Seville (IBIS), University of Seville, Seville, Spain

**Keywords:** primary care, health promotion, health behaviors and conduct, personal aptitudes, social determinants of health, emotional adjustment, self-care, qualitative research

## Abstract

**Introduction:**

Effective implementation of strategies to promote health and prevent noncommunicable illnesses requires a profound understanding of the interaction between the individual and society. This study brings to health research the consideration of psychosocial factors that influence the maintenance and change of health behaviors and conduct. From a primary care perspective, it is crucial to propose a biopsychosocial approach for the development of health promotion and self-care programs that embrace personal aptitudes as a relevant individual aspect.

**Objectives:**

To explore experiences related to personal aptitudes and personality traits that influence health behaviors and conduct, taking into account the social determinants of health, through a thematic analysis based on the capability-opportunity-motivation and behavior (COM-B) system.

**Methods and analysis:**

This qualitative research is carried out from a descriptive phenomenological perspective, based on 17 focus groups in which 156 people participated. Inductive and deductive analysis techniques were used following Lincoln and Guba’s criteria of methodological rigor. In addition to 7 different triangulations of analysts, 6 main categories were identified based on the COM-B system: psychological capacity, physical capacity, physical opportunity, social opportunity, reflective motivation, and automatic motivation. The importance of considering these factors to promote healthy behaviors was stressed.

**Discussion:**

This study examined how personal experiences related personal aptitudes and personality traits influence health behaviors and conduct in Spain. It was found that personality traits such as health literacy, self-efficacy, activation, and self-determination can influence the adoption of healthy behaviors. Likewise, the need for control, overthinking, and ambivalence made it impossible. Furthermore, social determinants of health and interpersonal relationships also play an important role.

**Trial registration:**

ClinicalTrials.gov, NCT04386135. Registered on April 30, 2020

## 1 Introduction

In the context of health, the complex dynamics of human behavior include both conscious actions and deeper, often subconscious, patterns of behavior. The term “Health Behavior” refers to the conscious actions that people take to promote or maintain their health, such as following a balanced diet or exercising regularly (1). On the other hand, the term “health conduct” includes a broader range of actions and behaviors, including aspects that may be conscious or unconscious, shaped by cultural, social and environmental factors. This may involve ingrained habits, social norms, or responses to external stimuli that affect health outcomes (2). In this study, we adopt a holistic approach, exploring the intertwined nature of “health behaviors and conduct,” recognizing their complex interaction with other influential dimensions internal to individuals, such as their personal aptitudes and personality traits. To understand how personal aptitudes influence health behaviors and conduct, we must first review the theories that underpin this assumption. Albert Bandura’s social cognitive theory emphasizes the role of self-efficacy and observational learning in shaping health behaviors and conduct, emphasizing the importance of personal aptitudes and environmental factors (3). Bandura postulates that people with a greater ability to adequately handle difficulties with a task or purpose (self-efficacy) are more likely to adopt and maintain health behaviors and conduct (4). Similarly, Irwin M. Rosenstock’s health belief model postulates that personality traits with greater susceptibility or severity create barriers that affect the use of health services (5). Rosenstock was one of the first to consider how personal aptitudes might influence health outcomes; however, his work was not closely related to health behaviors and conduct. Finally, Walter Mischel’s personality theory emphasizes the importance of differences in personal aptitudes and situational factors in predicting behavior (6). Mischel argued that aptitudes, together with motivational ability, were indispensable for acquiring and adapting to any health behaviors and conduct.

Understanding health behaviors and conduct is a complex task since they are influenced by a variety of factors, such as personal characteristics, mental health, and the social, environmental, and cultural contexts in which people live (7, 8). Personal and labor liabilities, socialization, care work, and labor overcharge, may significantly influence people’s health behaviors and conduct; therefore, a biopsychosocial focus seems to promise better results in health promotion programs and the population’s self-care (9). It has been identified little evidence about personal aptitudes influence on people’s health behaviors and conduct (10). For instance, a person with a higher level of self-control and personal health literacy may be able to maintain a healthy diet and a regular exercise regime (11). Likewise, people with a personality trait such as openness to experience or empathy may be more likely to participate in programs of preventive health, such as getting vaccinated, because they understand and value the impact that those behaviors could have on the health of others (12). A research study in Spain carried out by Armon, G., and Toker, S., examines how personality traits contribute to health behaviors and concludes that people with higher levels of responsibility and kindness take better care of their health. However, those with high levels of neuroticism tend to neglect aspects such as nutrition and physical exercise, and are more susceptible to the consumption of harmful substances (13). According to the World Health Organization, developing and strengthening personal abilities could be crucial to the development of a better health state among the population and to facilitating individual decision-making in health care (14). Successively, personal capabilities and health behaviors and conduct are conditioned by social determinants of health, which represent between 30 and 55% of health state results (15). Hence, they should be considered when it is intended to make efforts about knowledge and the promotion of personal aptitudes that foster or are related to positive conduct in health care in order to improve the adoption of healthy lifestyles and quality of life, just as the objectives of sustainable development dictate (16). Although social determinants in health may be fundamental to unraveling social and personal dynamics, these ones may be interrelated with external and internal factors with as many possible variables as branches of science itself (17–19). Thus, it is important to set study parameters in health determinants, such as social context, that can influence in factors like access to healthcare services, the availability of healthy food, life and work conditions (20), and people’s socioeconomic context that may influence access to these resources (21).

Behavioral science seeks to understand the psychological, biological, social, and environmental factors that influence behavior (5). According to health and social psychology, health behavior and illness are determined by aptitudes, beliefs, experiences, coping mechanisms, motivations, and attributions. The aforementioned results from social interaction, which may be reflected in the influence of experiences on risky behaviors. For example, a person who grows up in an environment where smoking is common and socially accepted may develop the belief that smoking is normal despite the health risks involved (22). These phenomena are accompanied by little-studied circumstances, such as the role of the family group in the promotion of individual health habits (23). And others are more widely studied, such as the socio-cultural factor, which determines the concept of health itself. For instance, the effect of social exclusion on the use of health services for certain marginalized groups (24). And the role of health literacy in experiences about health behaviors, which is rarely focused on the development of positive personal aptitudes (25).

Due to the lack of evidence to guide interventions related to personal aptitudes and personality traits that promote healthy behaviors among the population, we can appeal to the principles of behavioral change to generate ideas and strategies that promote positive health behaviors. Moreover, that helps us to intertwine the little-known dynamic between them (26). For instance, the capability-opportunity-motivation-behavior (COM-B) system identifies three factors that must be present for a behavior to occur: capability, opportunity, and motivation (27). Capability refers to the physical and mental capacity of an individual to participate in an activity. That includes the required knowledge and abilities to carry out a behavior (28). Opportunities are external factors that make behavior possible. This may include physical factors, like time and place, and social factors, like cultural norms and social signals. And motivation refers to conscious and unconscious cognitive processes that guide and motivate behavior (28). For instance, a study by the Department of Behavioral and Health Science in London, implementing the COM-B system, investigates conditions such as capability, opportunity, and motivational processes that increase personal protection behavior during the isolation phase of COVID-19 and analyzes personality traits such as agreeableness and conscientiousness. Furthermore, aiming to implement a program to decrease contagion (29). Another research study uses the COM-B system to improve adherence to self-care recommendations for heart failure. It also develops a theory-based intervention that demonstrates the need to include changes in health literacy and social support behaviors in the studied population (30). These studies make it more interesting to inquire about which behavioral techniques are implemented to improve the population’s prevention and self-care behaviors. Additionally, this type of system is a framework widely used to determine better strategies for behavioral change interventions to be effective. As illustrated in a research conducted on women with gestational diabetes, after applying the COM-B system, it included the positioning of parent counselors, a diabetes nurse, and the integration of a post-partum monitoring phase in a prevention program, improving their results (31). Other studies demonstrate how the COM-B system can be applied to develop interventions for behavioral change based on the theory and as evidence to improve self-care and chronic health conditions, as well as to comprehend how people’s health literacy levels influence their health behaviors and conduct (32).

Studying and analyzing the different behavioral systems and social health determinants altogether provides a complete insight into health outcomes and the quality of life of the population. Deepening the relationship between personal aptitudes (the ability, innate capability, or acquired capability that a person has to perform certain tasks or activities) and personality traits (enduring characteristics that describe a person’s consistent behavior) that influence health behaviors and conduct, taking into account individual characteristics as well as social and cultural contexts. Hence, the aim of this research study is to explore experiences related to personal aptitudes and personality traits that influence health behaviors and conduct, taking into account the social determinants of health, through a thematic analysis based on the capability-opportunity-motivation and behavior (COM-B) system.

## 2 Materials and methods

### 2.1 Study design

The study employed descriptive phenomenology, rooted in the philosophical tradition of Husserl, to examine and understand participants’ human experiences (33). This descriptive phenomenological research immersed itself in the specific details of the participants’ experiences, engaging in the exploration of a phenomenon through the meanings attributed by the participants (34). This approach was chosen because of its suitability for exploring the essence of lived experiences in relation to health behaviors and conduct (35). Although we adopted the subjective experience approach characteristic of descriptive phenomenology, which refrains from prior assumptions and focuses on people’s actual experiences (36), we acknowledge that we employed certain analytic approaches aligned with a more positivist paradigm, such as double coding in the two types of analysis. These deviations are justified by our goal of describing participants’ experiences in relation to our research questions, and by our approach to initial thematic analysis results, which falls between positivist and subjectivist orientations, as suggested by Braun and Clarke (37). These are further detailed in our data analysis section.

### 2.2 Environment

This study was part of a mixed-methods project called DESVELA Cohort, and the results presented here correspond to the qualitative phase. All details were described in the published protocols (38, 39). This study was conducted on primary care and health promotion during the period 2021–2023 in different Primary Health Care Centers in Spain from 8 Autonomous Communities (AACC; Catalonia, Euskadi, Castilla y León, Aragón, Galicia, Balearic Islands, Castilla la Mancha, and Andalusia).

### 2.3 Sampling

The power of the sample in this qualitative research did not depend on the size but rather on the representativeness of the discourse. This was the result of the combination of homogeneity and heterogeneity criteria and reflected maximum diversity and discursive plurality in relation to the phenomenon studied (Table 1) (40, 41).

**Table 1:** Sociodemographic data of focus group participants.

Variables	Sample	Percentages		Urban population	Rural population
**Age**					
Young adults (18 to 39 years)	17	10,90%		5,77%	5,13%
Middle-aged adults (40 to 49 years)	36	23,08%		12,18%	10,90%
Mature adults (> 50 years)	103	66,03%		35,26%	30,77%
**Sex at birth**					
Male	70	44,87%		25,00%	19,87%
Femenino	86	55,13%		28,21%	26,92%
**Level of education**					
Without studies and others*	6	3,85%		2,56%	1,28%
Elementary School	28	17,95%		7,69%	10,26%
High School	75	48,08%		28,21%	19,87%
University / Superior	47	30,13%		14,74%	15,38%
**Monthly household income level**					
No answer	7	4,49%		0,64%	3,85%
Less than 500€	2	1,28%		0,64%	0,64%
From 501 to 1,500€	43	27,56%		14,74%	12,82%
From 1,501 to 5,000€	96	61,54%		35,26%	26,28%
More than 5,000€	8	5,13%		1,92%	3,21%
**Health status**					
Excellent	25	16,03%		11,54%	4,49%
Very good	33	21,15%		13,46%	7,69%
Good	56	35,90%		14,74%	21,15%
Regular	29	18,59%		7,69%	10,90%
Bad	13	8,33%		5,77%	2,56%

*Without studies and others* only 1 participant in the sample did not know how to read or write, but this did not represent a problem for participating in the focus group.

Homogeneity criteria:

Determined by AACC and rural and urban population.

Heterogeneity criteria:

- Gender (sex at birth).
- Health status determined by the question, “How is your general health?”
- Income level determined by the question, “What is the approximate monthly income of your household?”
- Education level determined by the question, “What is your level of education?”

Other variables of the item without studies and others were cannot read or write. No studies, but can read and write.

In each CCAA, at least two Focus Groups (FG) were established in both rural and urban areas. Each CCAA selected the participants, taking into account heterogeneity and homogeneity criteria within the sample, and finally, a total of 17 FGs were formed, with an average of 10 participants per FG.

### 2.4 Participants and recruitment

Participants were invited to join in the FGs during the quantitative phase by means of a written request in the form of an informed consent. Subsequently, by telephone invitation, they were provided with detailed information on the purpose and conditions for participating in the qualitative study, as well as their right to withdraw at any time.

A total of 2.135 people expressed interest in being interviewed. A protocol was established to contact by telephone an average of 20–25 people for each CCAA. A total of approximately 340 people were invited to the FGs; the rest of those invited did not attend due to not being able to coordinate the itinerary, some because they were in charge of taking care of minors, and others due to a lack of time. Finally, 156 people were interviewed, forming 17 FGs. Participants had a range of 40 years of age and a standard deviation of 11.68; 86 were women and 70 were men (Table 1).

### 2.5 Information generation techniques

The study was based on FG, which involved the participation of several people in a single group interview (41) (Supplementary material 1). As part of the objective of a FG, we fostered interaction by sharing experiences and creating synergy among the participants through an interactive format composed of 3 dynamics that sought to encourage participation and avoid biases like social desirability.

The 17 FGs were video and audio recorded, and literal transcription was made. They were conducted in a relaxed and free-expression environment in an accessible and conformable place that allowed for an adequate time of approximately 90 min. A person with previous experience and/or training moderated participants’ dynamics during the FGs. In addition, another person observed the process and took field notes, providing support for her. Most moderators and observers of the FGs were women.

Several questions about health status, activation, and health literacy were asked (38). In order to execute the three dynamics, it was followed the Thematic Script of the Focus Group (TSFG) (38). The first written dynamic of personality traits: this dynamic was carried out with the aim that participants could anonymously describe their main personality traits and share an experience related to them that influenced in some way their health decision-making. The second dynamic of associative images was carried out with the intention of encouraging participation by means of an immediate reaction to images that were related to health behaviors and quality of life. These images facilitated discussion because they were created with the intention of showing the two extremes of health behaviors and conduct (healthy and unhealthy). The third dynamic of classification consisted of the interpretation and classification of images associated with healthy behaviors, which allowed us to know the importance that participants attribute to health behaviors and healthy habits, increasing the reflexivity of the discourse.

### 2.6 Data analysis

Two types of analysis were used to analyze the data. First, an inductive analysis with Colaizzi’s method was executed on 8 of the transcriptions of the study, with the aim of starting the triangulation of data with the other CCAA (42). The steps carried out were: (a) a general idea was obtained from each transcription by listening to the audios, watching the videos, and reading and rereading the transcripts. (b) Significant quotes were extracted, and a unit of meaning or representative code was assigned to each quote. (c) A formulation of general meanings was done. (d) Formulated meanings were organized into categories and subcategories. (e) The triangulation of analysts was initiated to discuss the emerging codes and categories. Here, each analyst previously carried out the steps (a–d) of the same material transcript chosen for the triangulation (Supplementary material 1.1). A second deductive or abductive analysis with the remaining nine transcripts using the Braun and Clarke method (37).

The executed steps were: (a) by reviewing, reading, and rereading, a category familiarization of the existent categories was created as a result of the first analysis in the 8 triangulations carried out previously. (b) An initial reading of the interviews was conducted, keeping in mind the existing categories and the units of meaning or codes linked to each category and subcategory. (c) A systematic coding was performed, extracting quotations that were related to the categories using the ATLAS.ti program (43). (d) The categories resulting from the inductive and deductive analyses were compared. (e) A thematic analysis based on the COM-B system was performed with the objective of organizing the categories resulting from the two analyses. Then the results were interpreted to find how the categories were related to the research objectives. (f) In order to report the results, by selecting the most illustrative quotes and the most eloquent categories, the research report was written. Saturation was achieved in terms of sufficiency thanks to the fact that a theoretical sampling was carried out that allowed enough data to develop a rich and detailed understanding of the study phenomenon. Through double codification and seven triangulations of the seven CCAAs performed, the results were verified. Equally, co-occurrence analysis with the most significant codes shows several codes with greater rootedness (44).

### 2.7 Rigor

The methodological rigor criteria of Lincoln and Guba (45) were followed. The credibility of the study researchers was ensured through their participation in the project of a multidisciplinary team from the *Fundació Institut Universitari per a la recerca a l’Atenció Primaria de Salut Jordi Gol I Gurina* and the credibility of the study were achieved thanks to an exhaustive work of several triangulations between researchers. By creating a detailed description of the discourses of participants and the study context, the transferability of the research was guaranteed. It was explained and justified in detail by the study method in compliance with dependability.

Moreover, a pilot test was performed following the TSFG; this pilot test was video recorded and shared with the other team members and those responsible for conducting the FGs. The pilot test demonstrated that the written and visual dynamics helped to encourage participation in the FG and avoid social desirability bias. The results were returned to participants as part of the transparency and rigor of the study, and the investigators have demonstrated honesty throughout the research process. This article fulfilled the standards for reporting qualitative research and also strictly followed the recommendations of the Consolidated Criteria for Reporting Qualitative Research (COREQ) (46) (Supplementary material 1).

## 3 Results

**Figure 1:**
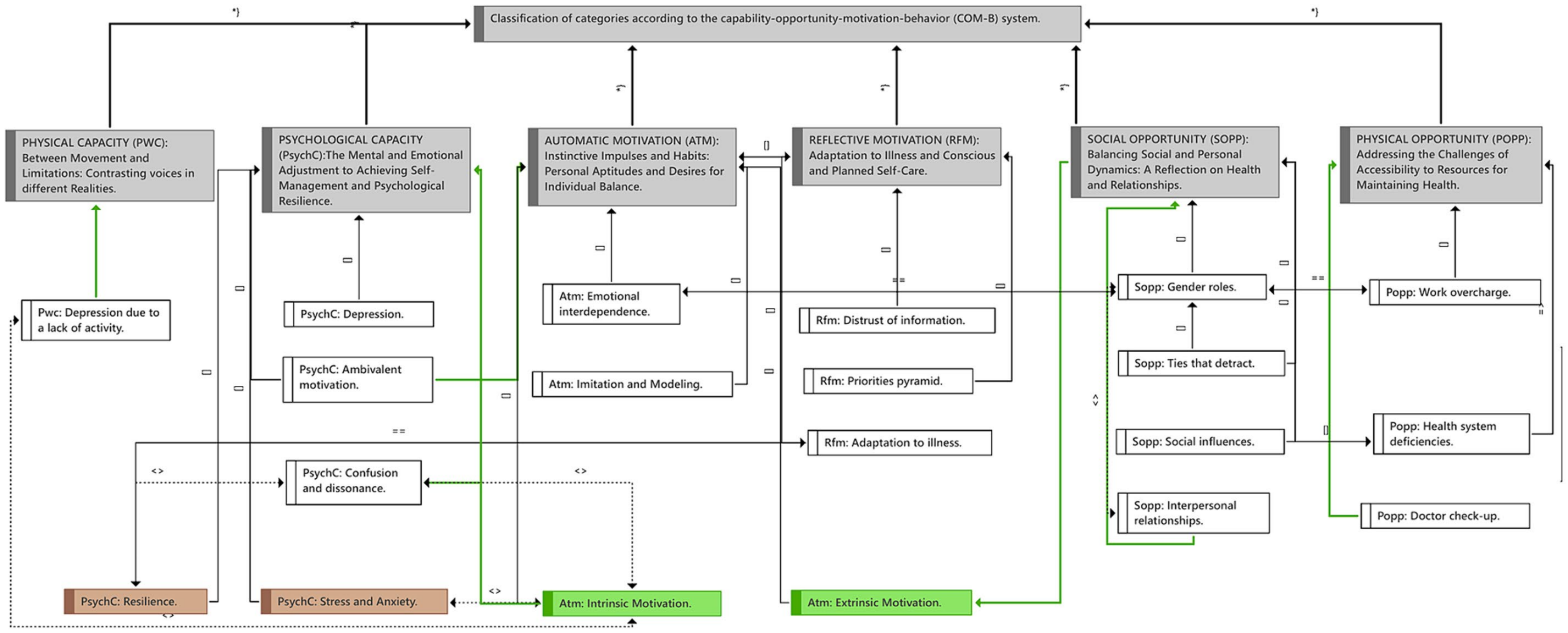
Summary of the resulting categories according to the capability-opportunity-motivation-behavior (COM-B) system, the codes with the highest rootedness (number of citations that have been coded by that code), and their relationships with each other. *Meaning of the Relationships: Contributes to (→), it is part of ([]), is associated with (═ ═), is cause of (═ ›), it is property of (*}), contradicts (‹›).

As a result of the inductive and deductive analysis processes, along with analyst triangulations, 6 main categories were developed. All of this is based on the COM-B system. **1.** Psychological Capacity: The Mental and Emotional Adjustment to Achieving Self-Management and Psychological Resilience; **2.** Physical Capacity: Between Movement and Limitations: Contrasting voices in different Realities; **3.** Physical Opportunity: Addressing the Challenges of Accessibility to Resources for Maintaining Health; **4.** Social Opportunity: Balancing Social and Personal Dynamics: A Reflection on Health and Relationships; **5.** Reflective Motivation: Adaptation to Illness and Conscious and Planned Self-Care; **6.** Automatic Motivation: Instinctive Impulses and Habits: Personal Aptitudes and Desires for Individual Balance (Table 2; Figure 1; Supplementary material 1.2).

**Table 2:** Categories and main codes, extracted from the research according to the capability-opportunity-motivation-behavior (COM-B) system.

COM-B topics	Categories	Most relevant codes
**CAPABILITY** According to the capability-opportunity-motivation-behavior (COM-B) system, capability is an attribute of a person that, together with opportunity, makes possible or facilitates a behavior.	**PSYCHOLOGICAL CAPACITY** **The Mental and Emotional Adjustment to Achieving Self-Management and Psychological Resilience.** Refers to the psychological ability to participate in activities from a mental perspective. It reflects participants’ ability to adopt health behaviors, such as internal struggle in the face of change and management of ambivalence.	Stress and Anxiety. Self-determination and Willingness. Self-efficiency. Mental resources. Depression. Confusion and dissonance. Health decision-making. Ambivalent motivation. Resilience.
	**PHYSICAL CAPACITY** **Between Movement and Limitations:** **Contrasting voices in different Realities.** Involves the physical and musculoskeletal abilities necessary to engage in health behaviors and conduct such as balance, dexterity, and autonomous mobility. It reflects the difficulties faced by some participants who can fully enjoy good physical capacity for the acquisition of behaviors.	Sport activities. Mobility limitation. Depression due to a lack of activity.
**OPPORTUNITY** According to the capability-opportunity-motivation-behavior (COM-B) system, opportunity is an attribute of an environmental system that, together with capability, makes possible or facilitates a behavior.	**PHYSICAL OPPORTUNITY** **Addressing the Challenges of Accessibility to Resources for Maintaining Health.** Related to environmental factors that influence behavior, such as financial resources, access to health services, and the immediate physical conditions of the participants.	Work overcharge. Doctor check-up. Health system deficiencies. Lack of financial resources. Food security.
	**SOCIAL OPPORTUNITY** **Balancing Social and Personal Dynamics:** **A Reflection on Health and Relationships.** Involves social factors that affect behavior, such as cultural norms, social influences, and peer pressures. Connected to the sexual division of labor in terms of gender roles and the influence of interpersonal relationships on participants’ health decisions and the implementation of healthy behaviors.	Gender roles. Social influences. Interpersonal relationships. Ties that detract. Active social life. Illness equals loss. Local context.
**MOTIVATION** Based on the capability-opportunity-motivation-behavior (COM-B) system, motivation is a set of mental processes that energize and direct behavior.	**REFLECTIVE MOTIVATION** **Adaptation to Illness and Conscious and Planned** **Self-Care.** It entails processes of conscious thinking, conscious decision-making, and advance planning by participants in the proper use of information and health literacy.	Priorities pyramid. Adaptation to illness. Activation. Distrust of information. Health experiences.
	**AUTOMATIC MOTIVATION** **Instinctive Impulses and Habits:** **Personal Aptitudes and Desires for Individual Balance.** It refers to habitual, instinctive, and affective processes, including the desires and habits of participants. Besides the influence of other people on their health decisions.	Extrinsic Motivation. Intrinsic Motivation. Happiness Habits. Emotional interdependence. Family Motivation. Imitation and Modeling. Personal aptitudes related to health decisions. Positive and negative aptitudes.

### 3.1 Psychological capacity: the mental and emotional adjustment to achieving self-management and psychological resilience

In this category, the discourses reflected the psychological and mental capacities that the participants used to adopt and maintain healthy behaviors, such as resilience, self-efficacy, self-determination, and critical thinking employed for proper health literacy. Furthermore, the challenges they faced while trying to implement these behaviors, such as managing depression, and the internal struggle in the face of change. Having the ability to manage problems was described as a criterion that could help in the adoption of some healthy habits like exercising and balanced eating. Resilience, positivity, assertiveness, rationality, and temperance were mentioned as coping tools by most of the participants who had serious health issues that prevented them from practicing healthier behaviors. Furthermore, they recognized that some aspects of their personalities, like being nervous and demanding at the same time, could cause anxiety or depression.

*You do not choose your health status, but you do choose how to face it. You can be happy, if you have a positive aptitude and do not give up no matter how bad the situation is. You will surely be happier. [Rural FG in Catalonia].*

Participants discussed the internal struggle between wanting to change and resisting it; some participants addressed this as an internal conflict experienced when trying to change habits but falling to do so. Most of the participants declared having the mental capacity and the motivation to implement healthier health behaviors and conduct but, in the end, not doing so. In addition, they cited a lack of willpower as the cause of this outcome and, in other cases, as a necessary criterion for quitting smoking and drinking alcohol.

*Let us see… I can understand that tobacco is bad, that alcohol is bad, and that everything in excess is bad. Now, if all of our lives we are in the phase of: do not eat this for cholesterol, do not eat that for sugar. I ask myself, What are you doing in this world? You have to do something! Because there comes a time when you say, What do I do? There is a proverb that says: “Whoever that neither smokes nor drinks wine is led by the devil in another way.” So you have to do something! [Urban FG in Galicia].*

Participants mentioned being conscious of how certain aspects of the personality affected their mental health. Aspects such as the need for control and overthinking were rated as negative personality traits that led to episodes of stress. However, they were mostly optimistic and declared having the capacity to manage negative emotions and maintain emotional adjustment. Even in the case of some participants with a diagnosis of schizophrenia or bipolar disorder, personality traits like being organized were manifested in time management and planning abilities to include health behaviors and conduct in their daily routine. Some participants were more open to performing activities they considered important to motivate them to improve their levels of activation, such as meditation and other activities related to sociability, creativity, and sports. In short, participants who declared themselves to be more extroverted and sociable were more open to doing this type of task, whereas other participants described themselves as more introverted and not very participative in these activities. Additionally, some mentioned personal aptitudes that they considered barriers to maintaining a more sociable lifestyle, such as being stubborn [“having little listening to the opinions of others”]. One of the participants confessed that he was sure that such a way of being made his daily activities more difficult. He expressed his desire for change and considered that a change of aptitude would bring him well-being.

*I always think that happiness is not a state but rather an aptitude, and I believe that it influences everything at all levels. Our emotional state, well, it is more than studied; it affects all our points of health status. I mean that I believe that how we take life will affect us emotionally and in terms of our health. [Urban FG in Andalusia].*

### 3.2 Physical capacity: between movement and limitations: contrasting voices in different realities

This category brought together the different experiences of participants in relation to their physical capacities to perform sports or movement activities, as well as the physical limitations of some participants as a consequence of chronic health issues or the perception of physical changes over time. In the same way, participants’ physical environment could influence, for instance, whether living in a rural area influenced the amount of physical activity they did or the excessive work conditions that led to a lack of time for regular physical activity. As well, opinions were expressed about taking physical activities to extremes. Most of the participants commented that an obsession with physical activity and appearance exists currently, especially among young people. One of the participants explained her experience with how her discipline led her to demand more physical activity from her body than it could tolerate.

*My perfectionist and disciplined way of being leads me to demand more physical activity from my body than my body often needs and can tolerate. I often somatize from this excessive activity in my body, and I am not able to take a break until my body warns me. [dynamics (WD)/Urban FG in Andalusia].*

Some participants commented on their preferences to practice physical activities such as pilates or yoga, among the most common ones, which were also related to the proper maintenance of mental health. They also included exercise, weight lifting, and walking in their routines and declared enjoying outdoor activities, mainly those living in rural zones who practiced activities such as mountaineering, hiking, and meeting more frequently to practice sports or even keep moving with field work. While those living in urban areas mentioned a lack of time or motivation to do so, they recognized the benefits of doing sports and wanting to improve their physical condition. Other participants noted a change in their level of physical activity over time. They noticed that before, they used to move more than now, and they perceived it as difficult to find the time or the willingness to do sports. One of the participants mentioned that having a dog at home created a need to increase his physical activity by having to take walks with his pet.

*With age, we lose something that is natural, and that is flexibility and elasticity. Of course, when you regain that, you become young again. You become young again, because, of course, I have right now the flexibility I had when I was 30 years old. Because those of us who have done sports and have not practiced much stretching, which is one of the things that is practiced a lot in yoga, get shorter with our muscles. [Urban FG in Andalusia].*

Many participants talked about their physical limitations, which prevented them from being more active, such as walking more, climbing hills, planting vegetables, dancing, and doing more things around the house, including enjoying some hobbies. Other participants mentioned having health problems, such as osteoarthritis and hernias, that impeded them from doing certain basic activities they enjoyed, but in general, they felt good. For the participants, being able to move around was key to their lives. Some who suffered a partial loss of mobility were satisfied with the possibility of being alive for many years to come. And others, despite not having physical difficulties or mobility problems, acknowledged that they did not engage in any physical activity out of lack of interest, laziness, or the excessive demands of their work. Not only working conditions but also workaholism by choice was recognized as a factor that prevented them from engaging in regular physical activity.

*It depends on the volume of work, but it also depends on the physical activity. Then I do everything wrong; if I have to do something from work, I put one thing before another, and in the end, when I realize it, I have messed up at the end. So, I see work addiction as a difficulty. [Urban FG in Andalucía].*

### 3.3 Physical opportunity: addressing the challenges of accessibility to resources for maintaining health

This category reflected the role of social determinants of health that were present in participants’ discourses. Such as experiences related to physical or material structures or resources in participants’ environment that had an impact on their healthy behaviors. Aspects such as the service in the healthcare system, the material resources available to guarantee food security, and the financial resources of the immediate physical environment. Their speeches expressed frustration at the lack of access to services they considered basic in primary care. Some criticized the inefficiency of emergency services and presented a general discontent with doctor-patient treatment, with an emphasis on psychological care.

They discussed the lack of accessibility to psychological services in the healthcare system and addressed the need for more mental health professionals and the need to improve mental health care in emergency services. One of the participants reported being on the verge of suicide at the time he asked his family doctor, but finally he had to pay for private psychological services due to the long wait for treatment. Several participants agreed on the difficulty of accessing psychologists through social security and the need to invest a lot of money in private treatment. In addition, a lack of friendly service from mental health professionals and the need for face-to-face medical appointments were mentioned.

*My current health state is bad. Bad, I am going through depression [the person seems a little bit down], and because of that, I asked you why there are not psychologists in the social security system. I have been in psychiatric treatment, and I asked the psychiatrist. Now, am I going to be sent to the psychologist? And she says no, and I ask, why? And she says, because there is none. That was the answer. Sure, nowadays, I know from my son’s experience that going to the psychologist is very expensive. And I do not understand why. But well. [Rural FG in Basque Country].*

Despite the general dissatisfaction, most of the participants emphasized the need for follow-up appointments, check-ups, and analysis by health professionals for the maintenance of proper health. For example, some people commented that they followed diets with nutritionists to lose weight, obtaining mostly good results. It emphasized the importance of prevention and the assessment of medical professionals in other fields. Some mentioned the lack of alternative treatment options, such as yoga or massages, and the lack of information on healthy habits offered in primary care. Other participants were very concerned about not having sufficient financial resources to have the opportunity to change their habits. One of the participants declared that despite working long hours for many years, he has not been able to manage his financial resources to strengthen his home and live peacefully.

*Sometimes I am very negative; apart from health, the thing that makes my life difficult is the financial status because I live in rented housing. [Urban FG in Castile and Leon].*

They also shared experiences related to food security, regarding concerns about food quality and prices. They mentioned the importance of healthy eating and the lack of time to prepare more elaborate and healthy food. Opinions on food prices were mentioned, with views aimed at comparing the opportunity to acquire food some years ago and now; they commented that currently healthy food is more expensive and difficult to access. In this regard, they expressed their opinion on how eating habits have changed, emphasizing the fact that food is more can-directed and preserved than homemade. Some participants commented that they considered eating home-grown food a better option. One of the participants gave his opinion on sugar consumption and mentioned the addition of sugar in many regular-consumption products, which he considered a controversy around healthy eating. Some participants stated that their family doctors have never forbidden them sugar, and for this reason, they did not consider it harmful to their health. They also mentioned their concern about an increase in illnesses, some of which were related to the quality of the current food. Most participants agreed with the current difficulty of finding good-quality food products at affordable prices.

*Now food is of less quality, our physical wear and tear is also less, and in the end all comes down to time: you get home after working the whole day and you do not mind frying some croquettes and having a salad, rather than make a good meal like those they used to prepare in the past. [Urban FG in Catalonia].*

### 3.4 Social opportunity: balancing social and personal dynamics: a reflection on health and relationships

This category included conditioning factors of the participants’ system of environment that had an impact on their healthy behaviors. Aspects such as cultural norms or social influences and pressures. For example, for participants, gender role experiences had an impact on the sexual division of work. Interpersonal relationships influenced palliative care for chronically ill participants, and some participants opined about the influence of media and social networks on their lifestyles.

Experiences about gender roles were mentioned mostly by participants who had experience with caregiving work with children or family caregivers. They reported how their dedication to their loved ones took time away from self-care activities such as medical check-ups or engaging in healthy behaviors like physical activity and socializing. This situation was described by some of the participants as very hard and exhausting, and some confessed that caregiving work generated constant stress and emotional pressure; however, they all agreed that the situation had no option for change due to the feeling of liability toward their children or relatives they take care of.

*But well, to improve my health status, I need my children to grow up a little bit and not be that demanding, well! Me, about exercising, well yeah, it is true that I could do something more, but well, I am currently implementing it in my life because before I did not do anything. I mean, since my children were born I have not done it, that is to say; I did things for them, but I did not do things for me. [Rural FG in Cuenca].*

They shared different experiences with their interpersonal relationships. But generally speaking, they concluded that problems with people in their close environment were emotionally and, in some cases, physically affecting, with some participants describing such situations as experiences with “toxic people.” Generally, discussions about the family led to more references to childcare and parenting models; the biggest challenges were managing caregiving responsibilities and shared concerns about ensuring a good future for their children. Some participants admitted to having difficulty relating socially as a result of suffering from a chronic illness, while others relied on their family or friends to find motivation in relation to caring for their illness. One of the participants stated that after confinement, they continued to feel a decrease in their social gatherings. Others expressed a sense of fear of returning to their encounters to go hiking or meet with friends and perceived that this caused a negative emotional impact and a decrease in their physical activity.

*In my life, there have been negative people, relatives, and the best I could have done was end the relationship. During…30 years, I have lived with the “oh, oh, oh” [expression of emotional pain], and at the end, I said, it is over! And it is the best I have done, even if it hurts, even when is a very close person. But at the end, it is better to put an end to the relationship with those negative people because they bring you emotional problems. [Urban FG in Andalusia].*

Gender roles continued to be part of the discussion, accompanied by experiences about themselves, their lifestyles, and their personal decisions. Some men, for instance, described themselves as “family providers and protectors,” to the extent that their only priority was to work for their families, and although they were aware of the need to take care of their own health and to attend regular medical controls, they stated that they did not set aside the time to do so. On the other hand, they discussed issues such as conflicts and a lack of communication within the couple, which they always attributed to causing low self-esteem, stress, and difficulty in achieving family reconciliation, even to the point where they declared that this affected their physical health.

*If you have an argument with a person or with your couple, I find myself emotionally unwell… It lowers my self-esteem; it lowers my defenses… It renders me more… I do not know, to think that I am not well! … Okay, psychologically, I am not well. And physically, it has an influence on me because when I have an argument with a person, personally, I think about it a lot. I mean, yes I leave too much room for it in my head and my defenses are immediately lowered. And, I am already sick after two days, or I have angina. The thing is, I somatized myself a lot. So, at the end, yeah, yeah, I find myself unwell. [Rural FG in Basque Country].*

### 3.5 Reflective motivation: adaptation to illness and conscious and planned self-care

This category described participants’ perspectives on factors that involved planned thought processes and self-evaluation that prompted them to engage in certain health behaviors and conduct, such as the importance of adapting their routines to their individual needs and evaluation habits related to health literacy. Moreover, the diversity of perspectives on what was critical to each of the participants as it related to their lifestyle habits. And some experiences about adapting to chronic illness through self-determination.

*I am a little bit hypochondriac about topics related to illnesses; you start searching through the internet and everything that appears, it happens to me, it gets me (expression to say that that stays in his thoughts). [Rural FG in Aragon].*

Participants reflected on the most important habits in their lives, emphasizing the importance of rest and nutrition, while some mentioned wellness activities, such as having time to relax and sunbathe. Personal hygiene and physical activity were mentioned as less important. In general, each person had his or her own priorities; nonetheless, they always emphasized what made them happiest. For some participants, the most important aspects of their lives were having a good family relationship, social life, and personal relationships. In addition, they mentioned some personal aptitudes that led them to adapt more easily to their routines, such as self-efficacy and activation.

*I am a very active person, and I always think about doing things. I always think that I have to do this and that, and I believe that that helps me to always be moving. [Rural FG in the Balearic Islands].*

Most of the participants reported going through very complicated health experiences, and others found themselves managing chronic illnesses for many years. In most cases, the participants have managed to adapt to the illness successfully to have a better quality of life. Some faced illness or the loss of a close family member. More than one participant mentioned that they were affected by a stroke, but they made an effort to keep busy with various activities, which helped them to maintain a positive aptitude. They learn to manage pain and sleep better. Even those who had a negative perception of their health status were generally optimistic about their recovery and progress. They reflected on self-awareness and their relationship to their own health, such as the importance of listening to their bodies and applying individually tailored health advice. Some participants described themselves as obsessed with their current lifestyles (vegan eating, high-performance sports), but allowing themselves to bend the rules from time to time.

*After an illness diagnosis with a poor prognosis, thanks to aptitude, curiosity to search, and perseverance, I got to know and obtain the best possible help and to be balanced with my emotional part. The rest did not depend on me. [Rural FG in Catalonia].*

Participants showed interest in consulting for information on integrative medicine and healthy eating. However, some were not very confident when relying on the information from the internet and felt that not all of it was accurate. Some participants planned activities that they considered emotionally and physically beneficial, such as practicing a physical activity every day (yoga, pilates). Many topics related to priorities in daily life and in life in general were discussed, such as finding a balance between enjoying exercise and relaxing on the couch. They valued illness prevention and related it to the ability to be responsible and stay well informed to make better health decisions.

*The Internet is such a thing… From the internet, I do not believe that much. Before you went to the library and got a book and checked the information, now everything is easier. [Rural FG in Galicia].*

### 3.6 Automatic motivation: instinctive impulses and habits: personal aptitudes and desires for individual balance

This category involved the instinctive processes associated with the desires and needs that drove the participants’ health behaviors. They talked about their motivations related to the drive for change. In some cases, this impulse arose from the example of a role model, in others from the advice or accompaniment of family or friends, and finally from the participants’ own self-determination to change.

For the participants who had experienced smoking cessation and excessive alcohol consumption, the need for family companionship was present, so their speeches reflected the importance of love, patience, and dedication in personal relationships and emphasized the relevance of keeping themselves happy every day to continue with the desire to not relapse. Some mentioned the feeling of urgency to be socially accepted, reflected in the experience of going so far as to drink or smoke just because of the need to fit in. Most participants were interested in sociability, in some cases stating that their social side was their motivating factor in maintaining good habits. Nonetheless, some experienced sociability from a negative perspective, surrounding themselves with people who raised concerns or increased personal problems. Some proximate influences related to healthy decision-making are also mentioned. For one, there was a need to please the family by cooking dishes that contained more hydrogenated fats, despite the desire to consume other types of food.

*I have not smoked for 22 years. Okay, I was a social smoker; I have to acknowledge it. Every weekend, two packs, every day, so, for example, I respect that others… although I do not mind if the smoke affects me, okay? [Rural FG in Basque Country].*

All of the participants described themselves, and some found a relationship between their personal aptitudes and their level of motivation to change. For example, some participants shared that they constantly suffered from feelings of worry and distress and that this did not allow them to go about their day-to-day lives as planned. These experiences were mostly related to problems with sleep habits and enjoyment of free time. In the first case, participants commented that overthinking led them to not being able to sleep enough or not having an optimal rest to start the routine, and in the second case, not being able to have free time to enjoy leisure or relaxation activities was linked to wanting to control everything. The need for control in all cases was an experience that led to nervousness and anxiety. Others showed an introverted personality that led them not to attend the doctor’s office because of embarrassment.

*Being a perfectionist makes me focus on small things without paying attention to other, more important things; being shy makes it harder for me to open up for help when I need it; being nervous makes it difficult for me to maintain calmness in the face of stressful situations; and sometimes I have seen myself overwhelmed [it expresses not being able to bear the situation]. Being responsible at the work and with my family makes that I prioritize other people over my own health and wellness. [Rural FG in Cuenca].*

## 4 Discussion

**Figure 2:**
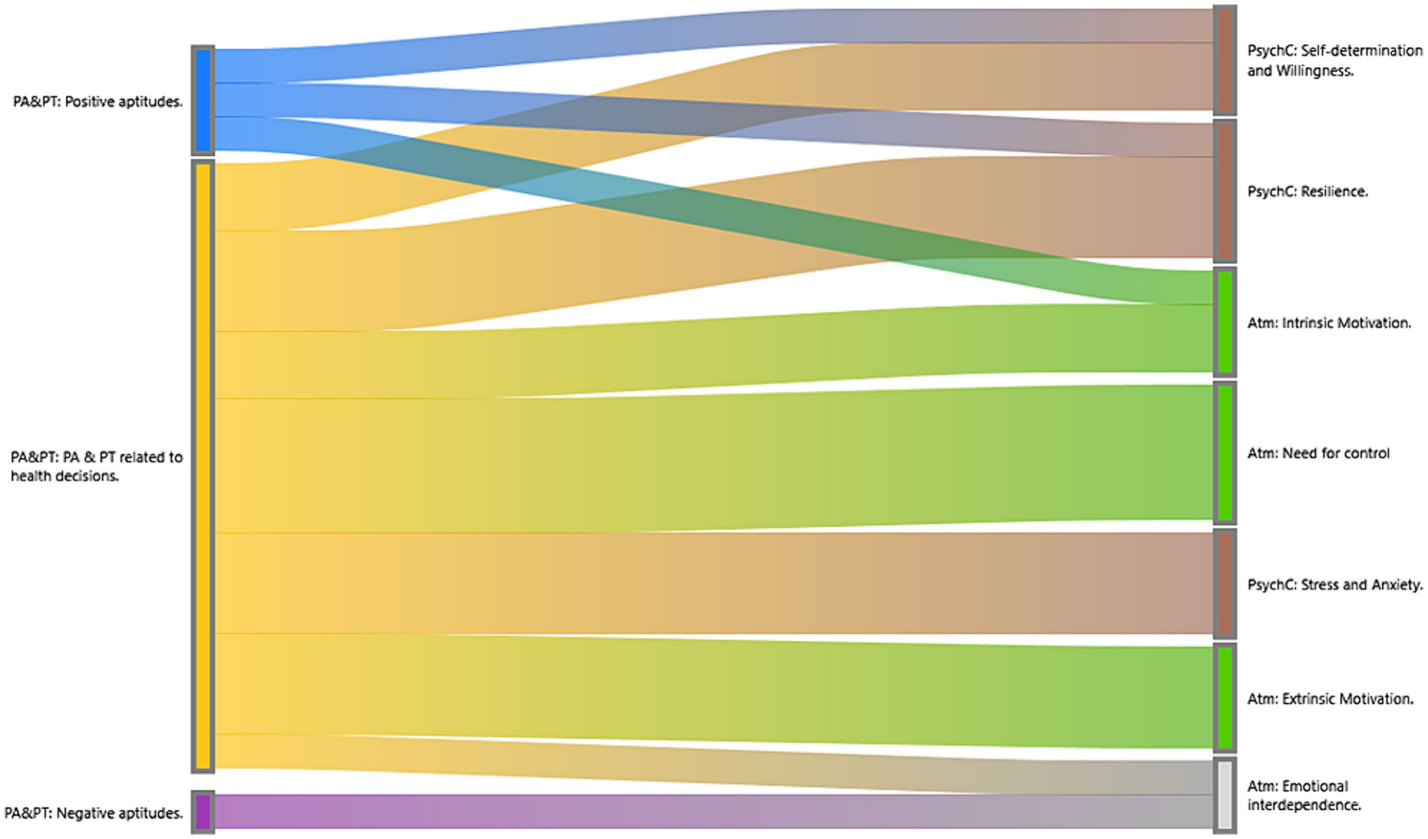
Sankey diagram: Relationship between personal aptitudes, psychological capacity, and automatic motivation. In the Sankey diagram, we can appreciate the co-occurrences of the codes or the relationship between them. Personal aptitudes and personality traits that participants described as tools to motivate change, such as self-determination and resilience, have a stronger relationship with and better self-perception of health and the acquisition of healthy behaviors. The strong relationship between the need for control and both intrinsic and extrinsic motivation is emphasized as a criterion for increased risk for unhealthy behaviors and how automatic urges are directly related to negative aptitudes.

The study delves into the experiences of the Spanish population regarding their personal aptitudes and health behaviors and conduct, where six main categories are identified that include psychological capacity, physical capacity, physical opportunity, social opportunity, reflective motivation, and automatic motivation. Based on our participants’ narratives, the main findings were related to personal aptitudes and personality traits that influenced psychological capacity and automatic motivation to change (Figure 2). This is relevant for the implementation of campaigns for the promotion and acquisition of healthy behaviors.

First, we will talk in terms of the capacity for change. Our results found that personal aptitudes such as resilience and personality traits such as self-determination were related to the psychological capacity needed to adopt healthy behaviors, and that the motivation required to maintain them came from intrinsic causes related to optimism and responsibility. Some related studies suggest that personal aptitudes such as extraversion and responsibility can improve people’s psychological capacity and academic performance, which could help in the adaptation of health-promoting behaviors and lead to a higher level of health literacy (47, 48). Other studies support the idea that resilience is closely attached to psychological capacity, as resilience is the ability to adapt positively to adverse or stressful situations (49). In our study, resilience was manifested through positivity, self-efficacy, rationality, and self-awareness as ways to manage stress and maintain good mental health. In conjunction, participants mentioned that practicing sports and seeking professional help were strategies to maintain resilience and mental well-being. In conclusion, the relationship between resilience and psychological capacity has positive results for our participants’ health. However, in contrast, we concluded that stress, anxiety, and the need for control negatively influenced their level of resilience, affecting both capacity and level of motivation in light of change.

In our study, the most common mental challenges in trying to adopt healthy behaviors, according to our participants, included the internal struggle between wanting to change and resisting change associated with acquiring habits and overcoming addictions, making conscious and informed health decisions, and persistent stress in different aspects of their lives. Other studies agree that people use a variety of strategies to maintain motivation and overcome resistance to change, including seeking emotional support, seeking professional help to manage stress and maintain good mental health, and making informed decisions about health (50, 51). Nevertheless, our study showed that these strategies are not feasible for everyone and revealed a lack of mental health support (a shortage of mental health professionals in the public sector and long waits for psychological care). Other studies confirm the importance of promoting the ability to adapt and maintain a positive aptitude in the face of mental and emotional adjustment, which can contribute to better stress management, the maintenance of healthy social relationships, and informed health decision-making (52).

The ability to solve problems, adapt to individual needs, manage time, and plan healthy activities is fundamental to the adoption of healthy habits (53). Several studies focus on the most beneficial health behaviors and advice on how to promote healthy habits, but they do not go into depth in terms of the personal aptitudes and individual capabilities of people, which could help not only to acquire but also to maintain over time the change in healthy behavior (42, 43, 54).

According to the COM-B system, at least two of the three parameters must exist for behavioral change to be possible (capability, motivation, and opportunity). That led us to understand why, in our results, we found a close relationship between psychological capability and automatic motivation (Figure 2). Although in other studies automatic motivation is considered to come exclusively from internal drives and instincts, our results revealed that it was influenced by extrinsic causes, which are mainly related to experiences with family, friends, or role models. Other studies show that poor social relationships (lack of bonding, social support, and social stress) increase cardiovascular risk, particularly with the cumulative impact of social factors, demonstrating the impact of personal relationships on health (55).

It is appropriate to continue to investigate the factors that influence the adoption of healthy behaviors and to distinguish the risk factors from individual and social criteria. Our participants described how they perceived the impact of their social and personal relationships on various aspects of their emotional and physical care. In particular, they emphasized partner and work relationships as influential factors in their health decision-making. Other studies show that positive interpersonal relationships and emotional support are fundamental tools for facing difficulties, especially in people suffering from a chronic illness, which was also evidenced in the well-known effect caused by the COVID-19 confinement stage (56). Other studies have found that the development of healthy relationships and the adequate management of emotional dependence contribute to a better perception of health and greater adherence to treatment (57). A variety of studies find that the implementation of group therapeutic tools, psychological counseling, and training in communication aptitudes and personal conflict resolution are essential to improving people’s quality of life (58, 59).

In terms of opportunity, according to other studies, work overload affects people’s physical opportunity by limiting their capability to engage in healthy behaviors, mainly due to a lack of time as a result of overwork (60, 61). In our study, participants mentioned that work overload prevented them from participating in physical activities or enjoying hobbies, as well as preparing healthier and more elaborate meals. Additionally, it generated stress and exhaustion, which in turn affected people’s capability to maintain an active lifestyle. Some of our results evidenced the lack of physical and cognitive opportunities in patients with chronic illnesses, which produced undesirable effects of the treatment and altered their daily routines. Nonetheless, they also demonstrated coping and adaptation strategies in the face of illness.

Another of our major findings was reflected in the discourse centered on the sexual division of labor (62). Our participants talked about how gender expectations had an impact on care and the acquisition of health behaviors and conduct. Some studies agree with our results, for example, that gender roles affect health decisions related to the distribution of responsibilities in the household and access to health services (63, 64). Our participants pointed out that gender role expectations, namely the assumption that women should assume the care of dependents, were associated with reduced opportunities to access health services and to engage in healthy behaviors such as physical activities and social meetings. Furthermore, it was observed that women may be stigmatized by their partners as having poor emotional control. Finally, through associative imagery dynamics, women were shown to be more concerned about their physical appearance and more involved in activities related to health care, such as practicing yoga, consulting nutritionists, or attending emotional support groups. Other studies reflect the sexual division of labor by showing how gender roles can limit the time and energy available for self-care and health care (65).

**Figure 3:**
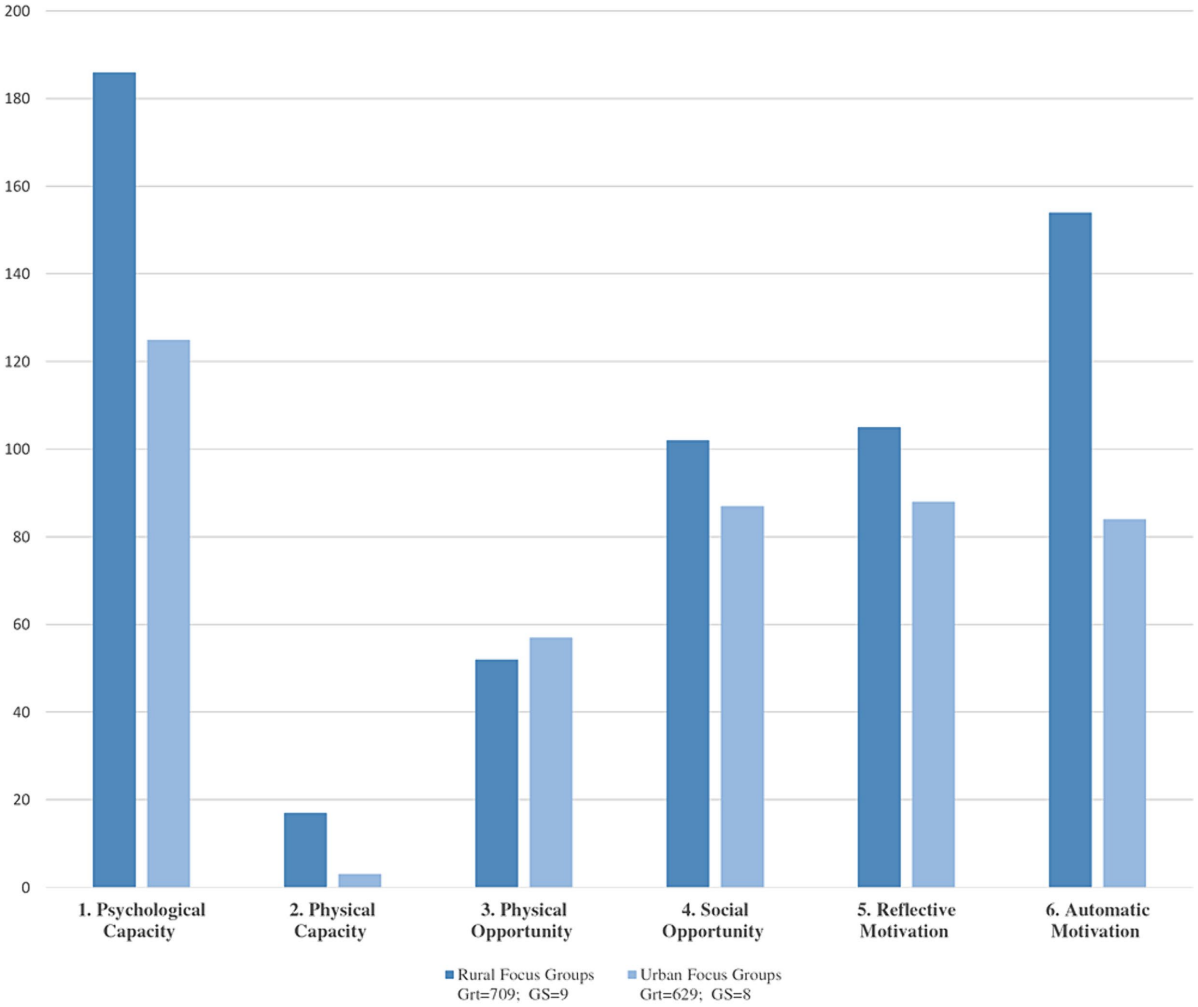
Diagram of code analysis document of the categories according to urban and rural area. Code Analysis-document: Comparison of the 17 urban and rural focus groups. Y: represents the number of codified quotes. X: represents category according to area. Grt = Is the number of total coded citations in the 17 focus groups by area. GS = Number of documents compared in each area.

Some of our strengths lie in the opportunity to carry out the study in an extended way throughout a large part of the Spanish territory. It is crucial to recognize that in qualitative research, the representativeness of the participants’ discourse is fundamental, not the size of the sample, as emphasized by numerous studies in the field (66, 67, 68). Sample size, considerably larger than in other similar studies (156 participants), is due to purposive and theoretical sampling, which ensured the relevance of the participants’ experiences in relation to the object of study, allowing us to oversaturate the codes, a crucial aspect in qualitative research (Figure 3). The semi-structured interview design also facilitated the deepening of the lived experiences. In summary, we believe that both discursive variability and careful selection of participants were crucial. Even with a smaller number of participants, we would have achieved data saturation in our study. Some of our limitations are based on the breadth of topics to be addressed, as in the case of the determinants of health, which are so broad and coarse that we were unable to cover them all in depth in this article. Another possible difficulty may be having the intention of addressing a variety of dynamics in the FG, as happened in some cases where the moderator felt the need to hurry and interrupt the fluid dialog of the participants, because of the eagerness to complete all the programmed dynamics TSFG (38). On the other hand, although in principle our intention in stratifying the sample by rural and urban areas was to find large differences, the comparison did not obtain more than a few discussions on feeding styles in rural and urban areas, but a large participation in both zones was evident (Figure 3). Finally, aspects to be improved in the field work were revealed for future studies using photo-voice or photo-elicitation. In some cases, the final objective was not achieved with such material because the participants did not focus their speeches on what the images transmitted to them, thinking from their personal experience, but rather limited themselves to describing the image they saw, moving away from the true objective of the dynamics. This could also be connected to the fact that only general training was given to the whole research team, and in some cases, the FG moderators were people who did not attend the training, which became evident when redirecting the participants’ dialogs to the objective of the dynamic.

## 5 Conclusion

In conclusion, the main findings of our research study reveal that personal aptitudes and personality traits can significantly influence the capability and motivation to change to implement a health behavior. Our participants remarked on the importance of personal problem-solving aptitudes, in addition to constantly practicing the search for tools of organization, time management, and planning to include healthy behaviors in their daily routine. They also emphasized the influence of interpersonal relationships on health decisions. In terms of health promotion programs, studies related to personal aptitudes and personality traits related to achieving the effectiveness of behavioral science theories have not yet been widely implemented. Thus, turning to high-quality intervention assessments to identify ways to improve the implementation of health behaviors and conduct that promote self-care is a viable option to recapture the essence of health promotion in the context of primary care.

In future research, it is suggested to carefully select the dynamics to be used in FG, limiting their number to one or a maximum of two per session to allow a more exhaustive exploration of the topics and to take greater advantage of each one. We conclude that the use of written dynamics to encourage the participation of people with introverted aptitudes is positive, since it reduces the social desirability bias. In addition, it is advisable to consult a professional expert in the use of images for behavioral dynamics, in order to generate discussion, ensuring that they are relevant and do not detract from the focus of the study. These recommendations will help to avoid shortcomings in the development of focus groups and will improve the quality of the research.

## Data availability statement

The raw data supporting the conclusions of this article will be made available by the authors, without undue reservation.

## Ethics statement

The studies involving humans were approved by the clinical research ethics committees from all the participant institutions: Fundació Institut Universitari per a la recerca a l’Atenció Primària de Salut Jordi Gol i Gurina (reference number 19/150-P); Comité de Ética de la Investigación con medicamentos del Área de Salud de Salamanca (reference number PI 2020 02424); Andalusian Ministry of Health, Spain (reference number:1260-N-20); Comité de Ética de la Investigación de medicamentos de Euskadi (CEIm-E; reference number: PI2020185); Hospital Virgen de la Luz Clinical Reasearch Ethics Comiitte, Cuenca, Spain (Reference number 2019/PI2119). Research Central Commission of the Primary Care Assistance Management, Madrid, Spain. (Reference Number 07/21); Comité de Ética de la Investigación de la Comunidad Autónoma de Aragón (reference number: PI20/302); Galician Ministry of health, Spain: high impact study authorization (Reference number: 2021/047). Collection, processing, communication, and transference of participants’ personal data will comply with the General Regulation (EU) on data protection (GDPR 2016/679) and the applicable national legislation, Organic Law 3/ 2018, of December 5, on the Protection of Personal Data. The studies were conducted in accordance with the local legislation and institutional requirements. The participants provided their written informed consent to participate in this study. Written informed consent was obtained from the individual(s) for the publication of any potentially identifiable images or data included in this article.

## Author contributions

YY-S: Conceptualization, Data curation, Formal analysis, Investigation, Methodology, Resources, Software, Validation, Visualization, Writing – original draft, Writing – review & editing. AB: Conceptualization, Investigation, Methodology, Project administration, Supervision, Validation, Writing – review & editing. DJ-C: Conceptualization, Investigation, Methodology, Project administration, Supervision, Validation, Writing – review & editing. RM-L: Conceptualization, Funding acquisition, Investigation, Resources, Supervision, Validation, Visualization, Writing – review & editing. PA-U: Writing – review & editing. OT-M: Writing – review & editing. IM-T: Writing – review & editing. UE-A: Writing – review & editing. FM-L: Writing – review & editing. XC-À: Writing – review & editing. EM: Writing – review & editing.

## DESVELA Cohort investigators

**Andalusia.** Universidad Loyola Andalucía: Emma Motrico, Irene Gómez-Gómez. Instituto de Investigación Biomédica de Málaga: Patricia Moreno-Peral, Sonia Conejo-Cerón, Juan Ángel Bellón. **Aragón.** Fundación Instituto de Investigación Sanitaria Aragón: Rosa Magallon-Botaya, Fátima Méndez-López, Alejandra Aguilar-Latorre, Maria Beltrán-Ruiz, Bárbara Oliván-Blázquez, Marta Dominguez-García, Maria Isabel Rabanaque Hernandez, Eva María Andrés Esteban. **Castilla la Mancha.** Centro de estudios sociosanitarios: Blanca Notario Pacheco, Montserrat Solera Martínez, Lidia Lucas-de la Cruz, Miriam Garrido Miguel, María Martínez Andrés, María Eugenia Visier Alfonso, Irene Marcilla Toribio. **Castilla y León.** Unidad de Investigación de Atención Primaria de Salamanca. José A Maderuelo-Fernández, Leticia Sierra-Martínez, Olaya Tamayo-Morales, Miriam Daniela García-Cubillas, Ana B Castro-Rivero, María D Martín-Santos, Carmen Castaño-Sánchez, Luis García-Ortiz. **Catalonia.** Fundació Institut Universitari per a la recerca a l’Atenció Primària de Salut Jordi Gol i Gurina: Bonaventura Bolíbar, Ruth Martí-Lluch, Rafel Ramos, Marc Casajuana-Closas, Anna Berenguera, Constanza Jacques-Aviñó, Yudy Young-Silva, Lia Alves-Cabratosa, Lluís Zacarías-Pons, Anna Ponjoan, Eva Espigulé-Ribas, Francesc Ribas-Aulinas, Jordi Blanch, Èric Tornabell-Noguera, Anna Moleras-Serra. Parc Sanitari Sant Joan de Déu: Enric Vicens-Pons, Montserrat Gil-Girbau, Mari Carmen Olmos Palenzuela, María del Carmen Gallardo González, Mª Teresa Peñarrubia-María, Paula Arroyo-Uriarte. **Madrid’s community.** Centro de Salud Infanta Mercedes, Servicio Madrileño de Salud. Francisco Camarelles Guillem. **Euskadi.** Instituto para la Investigación Sanitaria Biocruces Bizkaia. Jose María Aiarzaguena, Álvaro Sánchez Pérez, Sandra Garcia-Martinez, Usue Elizondo Alzola, Mónica Miranda de la Maza, Ainhoa Abrisketa Ullibarri, Mikel Rueda-Etxebarria. **Galicia.** Instituto de investigación Galicia Sur: Mª José Fernández Domínguez, Sabela Couso Viana, Roberto Fernández Alvarez, Ana Claveria Fontan, Ana Isabel Castaño Carou, Clara González Formoso, María Victoria Martín Miguel, Clara Guede Fernández, Macarena Chacón Docampo. Illes Balears. Gerencia de atención primaria de Mallorca, Instituto de investigación sanitaria de las Islas **Balearic Islands.** Joan Llobera Cànaves, Caterina Vicens, Maria J. Serrano-Ripoll, Laura Gallardo-Alfaro, Oana Bulilete, Christian Jean-Mairet Soler, David Medina-Bombardó, T Coll Benejam, Joana Ripoll Amengual.

## References

[1] Ryan P. Integrated theory of health behavior change. Clin Nurse Spec. (2009) 23:161–70. doi: 10.1097/NUR.0b013e3181a42373 19395894 PMC2778019

[2] Navarro López V Benach de Rovira J. Desigualdades sociales de salud en España. Informe de la Comisión Científica de Estudios de las Desigualdades Sociales de Salud en España [social inequities of health in Spain. Report of the scientific Commission for the Study of social inequities in health in Spain]. Revista espanola de salud publica. (1996) 70:505–636. PMID: 9011370

[3] Kelly S Martin S Kuhn I Cowan A Brayne C Lafortune L. Barriers and facilitators to the uptake and maintenance of healthy behaviours by people at mid-life: a rapid systematic review. PLoS One. (2016) 11:e0145074–26. doi: 10.1371/journal.pone.0145074, PMID: 26815199 PMC4731386

[4] Ozer EM. Albert Bandura (1925-2021). Am Psychol. (2022) 77:483–4. doi: 10.1037/amp0000981, PMID: 35238591

[5] Kroke AM Ruthig JC. Conspiracy beliefs and the impact on health behaviors. Appl Psychol Heal Well-Being. (2022) 14:311–28. doi: 10.1111/aphw.12304 34435446

[6] Ayduk O Mendoza-Denton R. Walter Mischel (1930-2018). Am Psychol. (2019) 74:740–1. doi: 10.1037/amp0000471, PMID: 31545642

[7] Davis JS Banfield E Lee HY Peng HL Chang S Wood AC. Lifestyle behavior patterns and mortality among adults in the NHANES 1988–1994 population: a latent profile analysis. Prev Med (Baltim). (2019) 120:131–9. doi: 10.1016/j.ypmed.2019.01.012, PMID: 30660707

[8] Dray J Gibson L Clinton-Mcharg T Byrnes E Wynne O Bartlem K. Exploring support provided by community managed Organisations to address health risk Behaviours associated with chronic disease among people with mental health conditions: a qualitative study with Organisational leaders. Int J Environ Res Public Health. (2022) 19:1–15. doi: 10.3390/ijerph19095533 PMC9105164 35564928

[9] Campos-García A Oliver A Tomás JM Galiana L Gutiérrez M. Autocuidado: nueva evidencia sobre su medida en adultos mayores. Rev Esp Geriatr Gerontol. (2018) 53:326–31. doi: 10.1016/j.regg.2018.01.010, PMID: 30430996

[10] Culatta E Clay-Warner J. I’m an adult now: health risk behaviors and identifying as an adult. J Health Psychol. (2022) 27:3164–76. doi: 10.1177/13591053221086184 35422145

[11] Gacek M Kosiba G Wojtowicz A. Sense of generalised self-efficacy and body mass index, diet health quality and pro-health behaviors of nursing students and active professional nurses. Med Pr. (2023) 74:251–61. doi: 10.13075/mp.5893.01382 37966381

[12] Appelman J Liebst LS Lindegaard MR. How common are high-risk coronavirus contacts? A video-observational analysis of outdoor public place behavior during the COVID-19 pandemic. PLoS One. (2022) 17:1–7. doi: 10.1371/journal.pone.0265680 PMC8929547 35298564

[13] Armon G Toker S. The role of personality in predicting repeat participation in periodic health screening. J Pers. (2013) 81:452–64. doi: 10.1111/jopy.12021, PMID: 23126563

[14] Gopinathan U Buse K. How can WHO transform its approach to social determinants of health? BMJ. (2022) 376:1–5. doi: 10.1136/bmj-2021-066172 PMC9685518 35135754

[15] Monni A Scalas LF. Health risk behaviour inventory validation and its association with self-regulatory dispositions. J Clin Psychol Med Settings. (2022) 29:861–74. doi: 10.1007/s10880-022-09854-z, PMID: 35099679

[16] Álvarez MR Llorente AHA del Llano Señarís JE. Los determinantes sociales de la salud en España (2010-2021): una revisión exploratoria de la literatura. Rev Esp Salud Publica. (2022) 96:e066172. 35582978

[17] Mies VC. El género como determinante social de la salud y su impacto en el desarrollo sostenible. Univ Rev Filos Derecho y Política. (2022) 41:33–47. doi: 10.20318/universitas.2023.7412

[18] Bjørnerud Korslund S Hansen BH Bjørkkjær T. Association between sociodemographic determinants and health behaviors, and clustering of health risk behaviors among 28,047 adults: a cross-sectional study among adults from the general Norwegian population. BMC Public Health. (2023) 23:1–8. doi: 10.1186/s12889-023-15435-y 36949417 PMC10031176

[19] Charreire H Conti B Bauchard L Cissé NA Perignon M Rollet P. A natural experiment to assess how urban interventions in lower socioeconomic areas influence health behaviors: the UrbASanté study. BMC Public Health. (2023) 23:1–9. doi: 10.1186/s12889-023-15388-2 36922807 PMC10015725

[20] 74th World Health Assembly (WHA). How to address DSS according to the World Health Organization (WHO). (2022). Available at: https://www.who.int/initiatives/action-on-the-social-determinants-of-health-for-advancing-equity/operational-framework/member-state-consultation-on-draft-operational-framework-for-monitoring-social-determinants-of-health-equity (Accessed March 23, 2021).

[21] Galea S Abdalla SM Sturchio JL. Social determinants of health, data science, and decision-making: forging a transdisciplinary synthesis. PLoS Med. (2020) 17:10–2. doi: 10.1371/journal.pmed.1003174 PMC7289342 32525875

[22] Fermino RC. Physical activity and health: social psychology perspective. Behav Sci (Basel). (2023) 13:4–5. doi: 10.3390/bs13040286 PMC10135945 37102800

[23] Michaelson V Pilato KA Davison CM. Family as a health promotion setting: a scoping review of conceptual models of the health-promoting family [internet]. Vol. 16. PLoS One. (2021) 16:1–37. doi: 10.1371/journal.pone.0249707, PMID: 33844692 PMC8041208

[24] Morales E. Exclusión social. Referentes teóricos y ejes analíticos desde el enfoque psicosocial Social Exclusion. Theoretical References and Analytical Axes from the Psychosocial Approach Instituto Cubano de Investigación Cultural. (2021) 1–21.

[25] Walters R Leslie SJ Polson R Cusack T Gorely T. Establishing the efficacy of interventions to improve health literacy and health behaviours: a systematic review. BMC Public Health. (2020) 20:1–17. doi: 10.1186/s12889-020-08991-0 32605608 PMC7329558

[26] Kvillemo P Nilsson A Strandberg AK Björk K Elgán TH Gripenberg J. Mental health problems, health risk behaviors, and prevention: a qualitative interview study on perceptions and attitudes among elite male soccer players. Front Public Health. (2023) 10–1. doi: 10.3389/fpubh.2022.1044601 PMC9850108 36684906

[27] Viinikainen J Bryson A Böckerman P Kari JT Lehtimäki T Raitakari O. Does better education mitigate risky health behavior? A mendelian randomization study. Econ Hum Biol. (2022) 46:101132–4. doi: 10.1016/j.ehb.2022.101134, PMID: 35354116

[28] Dorney P Pierangeli L. A phenomenological study: student nurses’ perceptions of Care of the Dying in a hospice-based facility. J Hosp Palliat Nurs. (2021) 23:162–9. doi: 10.1097/NJH.0000000000000730 33633097

[29] West R Michie S Rubin GJ Amlôt R. Applying principles of behaviour change to reduce SARS-CoV-2 transmission. Nat. Hum Behav. (2020) 4-5:451–9. doi: 10.1038/s41562-020-0887-9 32377018

[30] Whittal A Störk S Riegel B Herber OR. Applying the COM-B behaviour model to overcome barriers to heart failure self-care: a practical application of a conceptual framework for the development of complex interventions (ACHIEVE study). Eur J Cardiovasc Nurs. (2021) 20:261–7. doi: 10.1177/1474515120957292, PMID: 33909892

[31] Murphy K Berk J Muhwava-Mbabala L Booley S Harbron J Ware L. Using the COM-B model and behaviour change wheel to develop a theory and evidence-based intervention for women with gestational diabetes (IINDIAGO). BMC Public Health. (2023) 23:1–19. doi: 10.1186/s12889-023-15586-y 37189143 PMC10186807

[32] Herber OR. Patients with heart failure: a study protocol for developing a theory-based behaviour change intervention using the COM-B behaviour model (ACHIEVE study). BMJ open. (2018) 8:e025907. doi: 10.1136/bmjopen-2018-025907 PMC6144404 30206096

[33] Laverty SM. Hermeneutic Phenomenology and Phenomenology: A Comparison of Historical and Methodological Considerations. Int J Qual Methods. (2003) 2:21–35. doi: 10.1177/160940690300200303

[34] Macias GF. Methodology for phenomenological and / or hermeneutical qualitative research. Latin American Journal of Psychology. Psychol LA J Mexico (2018). 17:22.

[35] Krebs M Ghada A. mployment of colaizzi’s strategy in descriptive phenomenology: a reflection of a researcher.Service-learning: motivations for K-12 teachers. Eur Sci J. (2006) 8:31–43.

[36] O’Brien BC Harris IB Beckman TJ Reed DA Cook DA. Standards for reporting qualitative research. Acad Med. (2014) 89:1245–51. doi: 10.1097/ACM.0000000000000388 24979285

[37] Braun V Clarke V. One size fits all? What counts as quality practice in (reflexive) thematic analysis? Qual Res Psychol. (2021) 18:328–52. doi: 10.1080/14780887.2020.1769238

[38] Young-Silva Y Berenguera A Jacques-Aviñó C Gil-Girbau M Arroyo-Uriarte P Chela-Alvarez X. Role of personal aptitudes as determinants of incident morbidity, lifestyles, quality of life, use of the health services and mortality (DESVELA cohort): qualitative study protocol for a prospective cohort study in a hybrid analysis. Front Public Health. (2023) 11:1–9. doi: 10.3389/fpubh.2023.1069957, PMID: 37361167 PMC10289184

[39] Ruth M-L Bonaventura B Joan L Maderuelo-Fernández José A Rosa M-B Álvaro S-P. Role of personal aptitudes as determinants of incident morbidity, lifestyles, quality of life, use of health services, and mortality (DESVELA cohort): quantitative study protocol for a prospective cohort study in a hybrid analysis. Front Public Health. (2023) 11. doi: 10.3389/fpubh.2023.1067249 PMC10325828 37427254

[40] Davoudi-Kiakalayeh A Mohammadi R Pourfathollah AA Siery Z Davoudi-Kiakalayeh S. Qualitative methods in health care research. Int J Prev Med. (2017) 8:101–7. doi: 10.4103/ijpvm.IJPVM_246_16 29291043 PMC5738786

[41] Parra JD. El arte del muestreo cualitativo y su importancia para la evaluación y la investigación de políticas públicas: una aproximación realista. Oper Dent. (2019) 25:119–36. doi: 10.18601/16578651.n25.07

[42] Praveena KR Sasikumar S. Application of Colaizzi’s method of data analysis in phenomenological research. Med Leg Updat. (2021) 21:914–8. doi: 10.37506/mlu.v21i2.2800

[43] Woods M Paulus T Atkins DP Macklin R. Advancing qualitative research using qualitative data analysis software (QDAS)? Reviewing potential versus practice in published studies using ATLAS.Ti and NVivo, 1994–2013. Soc Sci Comput Rev. (2016) 34:597–617. doi: 10.1177/0894439315596311

[44] Saunders B Sim J Kingstone T Baker S Waterfield J Bartlam B. Saturation in qualitative research: exploring its conceptualization and operationalization. Qual Quant. (2018) 52-4:1893–907. doi: 10.1007/s11135-017-0574-8 PMC5993836 29937585

[45] Morse JM. Critical analysis of strategies for determining rigor in qualitative inquiry. Qual Health Res. (2015) 25:1212–22. doi: 10.1177/1049732315588501 26184336

[46] Tong A Sainsbury P Craig J. Consolidated criteria for reporting qualitative research (COREQ): a 32-item checklist for interviews and focus groups. Int J Qual Health Care. (2007) 19:349–57. doi: 10.1093/intqhc/mzm042, PMID: 17872937

[47] Cheng C Bai J Yang CY Li M Inder K Chan SWC. Patients’ experiences of coping with multiple chronic conditions: a qualitative descriptive study. J Clin Nurs. (2019) 28:4400–11. doi: 10.1111/jocn.15022, PMID: 31408566

[48] Nuria M Del RL Gardeb RA. Relationship between personality traits, styles and learning strategies and academic performance in adolescents Spanish students. Estud Pedagog. (2022) 48:273–89. doi: 10.4067/S0718-07052022000100273

[49] San Martín-García J Perles-Novas F Ángel García-Martín M Canto-Ortiz J. Adaptation of the beliefs about well-being scale to the Spanish population. Annals of Psychology. (2021) 37:233–42. doi: 10.6018/analesps.3362412294

[50] Cabanyes TJ. Resiliencia: una aproximación al concepto. Rev Psiquiatr. Salud Ment. (2010) 3:145–51. doi: 10.1016/j.rpsm.2010.09.003 23446037

[51] Alfonso Garzón Castrillon M. Capacidad Dinámica de Adaptación1. Revista Científica “Visión de Futuro”. (2018). 22:163.

[52] Ruíz-Fernández MD Fernández-Medina IM Granero-Molina J Hernández-Padilla JM Correa-Casado M Fernández-Sola C. Social acceptance of death and its implication for end-of-life care. J Adv Nurs. (2021) 77:3132–41. doi: 10.1111/jan.14836 33755231

[53] Godoy D Eberhard A Abarca F Acuña B Muñoz R. Psicoeducación en salud mental: una herramienta para pacientes y familiares. Rev Médica Clínica Las Condes. (2020) 31:169–73. doi: 10.1016/j.rmclc.2020.01.005

[54] Boga SM Saltan A. Identifying the relationship among sleep, mental status, daily living activities, depression and pain in older adults: a comparative study in Yalova. Turkey J Pak Med Assoc. (2020) 70:236–42. doi: 10.5455/JPMA.301384, PMID: 32063613

[55] Serafini M Cuenya L. Motivación: un recorrido histórico y teórico de los principales marcos conceptuales. Rev Concienc EPG. (2020) 5:15–44. doi: 10.32654/CONCIENCIAEPG.5-2.2

[56] Seeman TE Gruenewald TL Cohen S Williams DR Matthews KA. Social relationships and their biological correlates: coronary artery risk development in Young adults (CARDIA) study. Psychoneuroendocrinology. (2014) 43:126–38. doi: 10.1016/j.psyneuen.2014.02.008, PMID: 24703178

[57] Jacques-Aviño C Medina-Perucha L Young-Silva Y. Narratives of gendered changes in health behaviours during confinement in Spain. Gac Sanit. (2023) 37:296. doi: 10.1016/j.gaceta.2023.102296 36921453

[58] Zanolari D Händler-Schuster D Clarenbach C Schmid-Mohler G. A qualitative study of the sources of chronic obstructive pulmonary disease-related emotional distress. Chron Respir Dis. (2023) 20:147997312311638–10. doi: 10.1177/14799731231163873 PMC10009049 36898089

[59] Dwarswaard J Bakker EJM van Staa A Boeije HR. Self-management support from the perspective of patients with a chronic condition: a thematic synthesis of qualitative studies. Health Expect. (2016) 19:194–208. doi: 10.1111/hex.12346, PMID: 25619975 PMC5055271

[60] O’Reilly P. Methodological issues in social support and social network research. Soc Sci Med. (1989) 26:863–73. 10.1016/0277-9536(88)90179-7 3287636

[61] Dolores Ruiz E Salazar Gómez JF Valdivia Rivera MJ Dolores Ruiz E Salazar Gómez JF. La motivación laboral y su relación con el desempeño laboral. Un estudio de caso. RIDE Rev Iberoam para la Investig y el Desarro Educ. (2023). 5:11. doi: 10.23913/ride.v13i26.1478

[62] Peña Ponce DK Toala Pincay MY Toala Pincay BA. Sobrecarga de trabajo: efectos sobre la productividad y calidad de vida. Theatr Rec. (2022) 29–40:121. doi: 10.26820/recimundo/6.(suppl1).junio.2022.29-40

[63] Yavorsky JE Kamp Dush CM Schoppe-Sullivan SJ. The production of inequality: the gender division of labor across the transition to parenthood. J Marriage Fam. (2015) 77:662–79. doi: 10.1111/jomf.12189 26430282 PMC4584401

[64] Saldívar Garduño A Díaz Loving R Reyes Ruiz NE Armenta Hurtarte C López Rosales F Moreno López M. Roles de Género y Diversidad: Validación de una Escala en Varios Contextos Culturales1. Acta Investig Psicológica - Psychol Res Rec. (2015) 5:2124–47. doi: 10.1016/S2007-4719(16)30005-9

[65] Yang M Roope LSJ Buchanan J Attema AE Clarke PM Walker AS. Eliciting risk preferences that predict risky health behavior: a comparison of two approaches. Heal Econ (United Kingdom). (2022) 31:836–58. doi: 10.1002/hec.4486 PMC9305924 35194876

[66] Berenguera Ossó A Fernandez De Sanmamaed Santos MJ Pons Vigués M. Observing and understanding. Recovering narrative in the Health Sciences. Jordi Gol Univ Inst Prim Care Res (IDIAP J Gol). (2014) 224:112–16. doi: 10.1016/j.gaceta.2019.06.011

[67] Nelson J. Using conceptual depth criteria: addressing the challenge of reaching saturation in qualitative research. Qual Res. (2017) 17:554–70. doi: 10.1177/1468794116679873

[68] Brooks J McCluskey S Turley E King N. The utility of template analysis in qualitative psychology research. Qual Res Psychol. (2015) 12:202–22. doi: 10.1080/14780887.2014.955224, PMID: 27499705 PMC4960514

