# Supplementary material for: Exploring personal aptitudes and personality traits that, together with social determinants, shape health behaviors and conduct: a thematic analysis based on the Capability, Opportunity, Motivation and Behavior (COM-B) change system
Source: Front Public Health. 2024 Jun 5;12:1387528. doi: 10.3389/fpubh.2024.1387528 (PMC11186474; doi:10.3389/fpubh.2024.1387528)
Supplement: Supplementary file 1 [file Data_Sheet_1.PDF]

Supplementary Material

Perceptions about beliefs and personal aptitudes in health behaviors: A thematic analysis based on the capability, opportunity and motivation (COM-B) behavioral change system.

Supplementary Data

1. COREQ (COnsolidated criteria for REporting Qualitative research) Checklist.

A checklist of items that should be included in reports of qualitative research. You must report the page number in your manuscript where you consider each of the items listed in this checklist. If you have not included this information, either revise your manuscript accordingly before submitting or note N/A.

Topic	Item No.	Guide Questions/Description	Reported on Page No.
**Domain 1: Research team and reflexivity**			
Personal characteristics			
Interviewer/facilitator	1	Which author/s conducted the interview or focus group?	
Credentials	2	What were the researcher's credentials? E.g. PhD, MD	6
Occupation	3	What was their occupation at the time of the study?	6
Gender	4	Was the researcher male or female?	6
Experience and training	5	What experience or training did the researcher have?	6
Relationship with participants			
Relationship established	6	Was a relationship established prior to study commencement?	6
Participant knowledge of the interviewer	7	What did the participants know about the researcher? e.g. personal goals, reasons for doing the research	4
Interviewer characteristics	8	What characteristics were reported about the inter viewer/facilitator? e.g. Bias, assumptions, reasons and interests in the research topic	4

**Domain 2: Study design**			
Theoretical framework			
Methodological orientation and Theory	9	What methodological orientation was stated to underpin the study? e.g. grounded theory, discourse analysis, ethnography, phenomenology, content analysis	4
Participant selection			
Sampling	10	How were participants selected? e.g. purposive, convenience, consecutive, snowball	4
Method of approach	11	How were participants approached? e.g. face-to-face, telephone, mail, email	4
Sample size	12	How many participants were in the study?	4
Non-participation	13	How many people refused to participate or dropped out? Reasons?	4
Setting			
Setting of data collection	14	Where was the data collected? e.g. home, clinic, workplace	4
Presence of non-participants	15	Was anyone else present besides the participants and researchers?	4
Description of sample	16	What are the important characteristics of the sample? e.g. demographic data, date	4
Data collection			
Interview guide	17	Were questions, prompts, guides provided by the authors? Was it pilot tested?	5
Repeat interviews	18	Were repeat inter views carried out? If yes, how many?	5
Audio/visual recording	19	Did the research use audio or visual recording to collect the data?	5
Field notes	20	Were field notes made during and/or after the inter view or focus group?	5
Duration	21	What was the duration of the inter views or focus group?	5
Data saturation	22	Was data saturation discussed?	5
Transcripts returned	23	Were transcripts returned to participants for comment and/or	6

1.1 Instructions for analyst triangulation.

From the qualitative coordination of the DESVELA project, we extend an invitation to participate in the triangulation of qualitative data. To do this, we attach the transcript of the triangulated interview, as well as this document detailing the step-by-step instructions on the activities to be carried out.

Study objective: To explore participants' experiences and beliefs about their health behaviors in relation to their personal aptitudes (activation, personality traits) and health literacy. Additionally, the specific objectives are:

-Understand the dynamics of health behaviors according to the quality of life and health status of the participants.

- Explore participants' opinions and experiences on health-related behaviors according to their different local, social, economic or educational contexts.

Triangulation carried out by the autonomous community:

1. Reading/Rereading and Familiarization Note. Write a note (memorandum) here with the main ideas extracted after reading the complete transcript of the assigned interview.

2. Coding: Read or listen to the interview as many times as necessary and assign codes to the most relevant dialogues. In the same interview with the Review/Comment function.

Note: Keep in mind that, although the codes can be interpretive, you cannot leave aside what the participant describes in his speech. When you finish, you must send the Word transcript with all the comments to be able to identify where each code comes from.

Example:

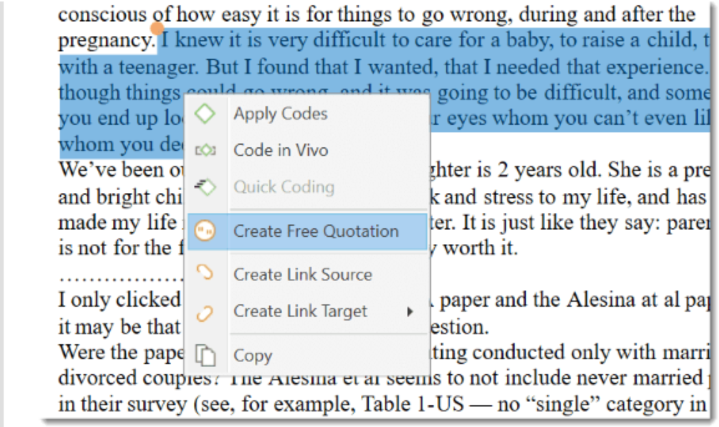

Once a quotation is created, you see a blue bar in the margin area and an entry in the Quotation Manager and the Document tree in the Project Explorer.

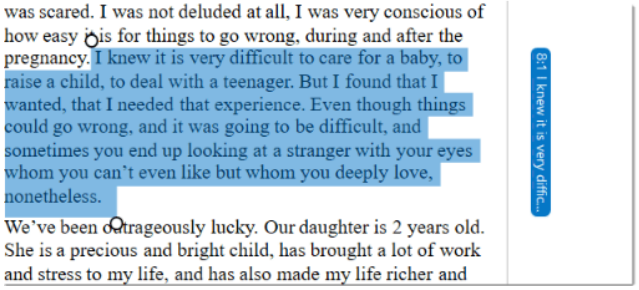

3. Generate initial categories. Table of categories, subcategories and codes (or initial thematic map) It consists of classifying the different codes into potential categories or themes. It may be helpful to use visual representations or tables.

Note: Review what you put in step 1, sometimes your intuition has already thrown up some categories☺.

CATEGORY	SUB CATEGORY	CODES	VERBATIMS

4. It is time to agree to a meeting with Catalonia, to compare the results through triangulation and reach an agreement on the categories.

Thank you very much for your dedication and effort to make this research possible!

1.2 Most representative quotes from the developed categories.

	Categories	Most representative quotes
capability	1<br><br>**Psychological capacity: The Metal and Emotional Challenge to Achieve Self-Management and Psychological Resilience.**	*"Obviously, my calmer personal attitude makes it better, otherwise I could be much more depressed. Or he [points to another participant] who has genetically more predisposition to have diseases, if he didn't have a good mood, he would have more! He would be depressed... He would probably have more problems. I believe that personal skills do help you manage... Of course they affect your health! Because it's you managing your boat. If you had another way of approaching it, you would probably be worse off."* [6:46 ¶ Rural GF in the Balearic Islands].<br><br>*"I, for example, the character. It has a lot of influence, because that need to do things quickly that I have sometimes makes me very nervous and there are times when my stomach gets very nervous and I notice it. But there are times when I try to relax, but I am very explosive, like champagne, boom! I get up and down, and in that circumstance I don't have a very good time. And I would really like to change that."* [74:48 ¶ GF Rural in Aragón].<br><br>*"I have bipolar disorder. So, for me, my bipolar disorder has been my greatest learning in life, because the experiences that I have had both in a reversal state, since I was little, as well as in states in which you think you are the king of the mambo and then you discover that no, there are many other mambo kings, because they help you grow. They help you, and force you to know yourself enough. To live with yourself first, learn to love yourself, and then go out into the world with your loved ones."* [9:33 ¶ GF Urbano in Andalusia].<br><br>*"Being more and more stressed as you grow older is a problem, even if it is the same, that is, I and another person can have the same problems, but one can manage them and the other cannot, and make them come out somewhere. Why do we all have problems? Of one importance or another, we all have problems. But, the way of managing them is different for each one"* [73:51 ¶ 1306GF Andalusia Urbano_2]<br><br>*"Each one of us has to learn, I believe... Each one finds potholes, stones on their way... I went this way, I like life... But then life gives you a stone, a wall, a hedge, and There is the learning of each one of how you overcome... And there are times that you are not able to use those personal tools that you have to overcome all that, but you learn. And that is very important."* [17:37 ¶ 514Urban Basque Country]<br><br>*"Now I have a knee problem... I have atrophic psoriasis, and there are times when it hurts... then it's like I don't feel like leaving the house, I've had a period of... which I know is harming me. And there are times when I go out, because my husband forces me, and in an hour I'm like the foxes [from the expression to be like the foxes. Very tired]... I'm going home... so it's a little strange, I can't stand it well, at home... notice that it has turned out differently... I don't know girl, I know I'm doing it wrong, now I've signed up for a gym to see if I can do something...but come on"* [3:20 ¶ 393 GF: Aragon Urbano].<br><br>*"I was always a very strong person, but the stroke broke me. In other words, I thought they had everything ahead of me, but they took me ahead of me. So that, well, it has totally conditioned my life. At work, at home, with the family... Everything. It has completely conditioned me. I have days when I can't do anything, I have days when I do something. At that moment I have surgery on both eyes and I don't see almost anything, and things like that. Yes, it is true that sometimes I go to the doctor, some new doctor and such, and he sees the history and asks me, "How are you?" And me, fine. And he tells me, well, you're great! But hey, in everyday life you eventually forget everything. All this. You try to be well every day and... Well, I think I'm used to drawing strength from where there is none. There are days and times when you are not well, and that's it, you don't think about when the next one is going to come. Oh well!"* [87:27 ¶ 182 – 194 GF: Galicia Urbano]<br><br>*"I have a problem too, and that is that every year I have an analysis done and every year I have a check-up with the doctor, and everything always comes out perfect. And you think: what do I have to change? If it looks good, right? I know that in the long run you are not okay, because this... is not good, in the long run a day will come that will say so much about eating is not good. I know this, but of course, you look at yourself, and the perfect cholesterol, the perfect glucose, everything perfect, and you think: No, no! And my wife scolds me, or she gets impatient, because she lives a healthier life, in quotes, because she goes to the gym, tries to eat healthier things, and her cholesterol levels are higher. And you think, and why?"* [6:21 ¶ 363 – 367 GF: Rural Balearic Islands]<br><br>*"What I'm saying is that I'm too lazy to exercise. I would like to exercise more than I do... and of course, I also get tired... well that... That... that's, that's a problem. I just don't know, I don't know if I can improve it."* [3:13 ¶ 303 – 311 GF: Aragón Urbano]<br><br>*"Well, look, for example, my experience tells me that I should eat healthier, why I should eat more fruit, it's true, because fruit... I should eat a lot of fruit, and also improve my meals because I'm a little messy, I don't know if it's the medication that affects or that... quit smoking, I should do it too... try to take better care of myself than almost all the doctors tell me... what happens is that my health problems overcome my spirit, that's why I have a difficult time finding that mental stability. body... it is the most complicated... because the mind tells you one thing, obviously it tells you many*

		things, because I am a very hard-working and very active person, and my head is ahead, many times it is ahead of everything and... yet my body ... then there is a confrontation... very complicated to manage... often very complicated."[3:29 ¶ 503 GF: Aragon Urbano].<br>*"Food, above all. It's been clapper for years and it hasn't. I am not capable of [expression of shame] I have no willpower to. Unfortunately... I really like it sweet, now not so much, because now I gradually manage to eliminate it a little, but in the morning, at 7 I have already eaten an ensaimada and a chocolate donut, for example. Now I no longer have to suffer for anything, now I have taken it off, lately, for the last two months, and instead of this, a piece of chocolate cake, *cacao, which is more... Which is 70 percent, It takes away your desire for sweets a little, because you have to go in the mornings... [*laughs] But it costs me a lot, a lot. I mean, it's... I try, but it's really hard for me."*[6:14 ¶ 443 GF: Baleares Rural].<br>*"The same thing happens to me, I say I'm going to do it and then I don't do it [do physical exercise] plus I take two steps there, I go up the stairs and I feel like it's hard for me. And I have to give up tobacco too."*[13:9 ¶ 174 – 194 in Cuenca_Rural_1]<br>*"The doctor knows me very well, and she already says, to keep you still, not even tied up! . I am a person obsessed with work, and I have to work. I don't care if it's in the company or at home. I have vineyards, I have everything, and I have to work, I can't stay still! It's something I'm not capable of. If I have to get up at five in the morning, I get up. I get up early every day, I get up at six every day, and at this time, in the village, on the weekend too. Even if I go to bed at one or two, it doesn't matter to me.*[3:94 ¶ 306 GF: Galicia Urbano]<br>*Being nervous doesn't help me because everything has to be done before yesterday (an expression to indicate that you want things for right now!) and I consider myself active but not constant, so I usually start things and continue whatever I want and never I finish them. That happens to me with the discipline of going for a walk, the fact is that when I walk I feel better, but I don't do it.*[D_Written ¶ Urban GF in the Basque Country]<br>*"This is here [points to head] Yes, the best solution is to consider it, you are never going to quit smoking, quit smoking two packs a day. That's impossible! One day you say: I don't smoke anymore. I left it 50 times eh, leaving it and starting again. But one day I said, I don't smoke anymore."*[14:18 ¶ 634 – 652 in Galicia_Rural] *"*<br>*"I was diagnosed with schizophrenia, I have had admissions, I have had outbreaks, as I always thought, I have nothing! and in the end I do take the medication and in the end I am achieving things that physically and intellectually, reached an average, I am not a brilliant mind, but my head does work"*[2:14 ¶ 191, GF: Catalunya Rural_2]<br>*"Selfishly to myself. Because only I know myself. Let's see that she could give me some advice, I receive it,... she analyzes it, but deep down I know what I am going to act and what I am going to do. I... and... I mean, and... I try not to fool myself. At the time of arrival, I know myself, I know what is good for me and... er... I am one of those who weigh things up. This yes, this no, this yes, this no. I grab the good, what I can and that's it. Selfishly, that. Myself."*[19:36 ¶ 209, GF: Cuenca_Rural_2]<br>*"The problem is how difficult it is to discern. Not finding information. That you can see things that you can think: I'm doing this because it's great for me, because I've read it in forty places and maybe not. It's probably not bad either, but it's not good either. And the information, right now we are extra... we can find it everywhere, on the internet... etc. The idea is to discern the sources, really, where they come from."*[9:26 ¶ 154, GF: Rural Basin_2]<br>*"Being extroverted and demanding at the same time is a difficulty that I have to work on regarding my mental health, since this dichotomy produces chronic anxiety."*[D.E_Catalunya Urbano Gàva]<br>*"Going through too much grief harms my health since it generates both psychological and physical discomfort in the end."*[D.E_Baleares Rural]
	**2**<br><br>**Physical Ability: Between Movement and Limitations: Contrasting Voices in Different Realities.**	*"I have summarized it with a single word, and I have called it "mobility". The most important thing in my life is that I can move. At the moment. I spent nine months in bed without getting up even to go pee, and when I started walking I said, damn, I'm fine. How splendid I am, right? And from here, well... Move forward, and everything else will come"[6:89 ¶ Rural GF in the Balearic Islands].*<br>*"I would like to be able to walk more, but because of my seven hernias I can't, I would like to do more things at home, gain weight in a bag, I would like to be able to grow vegetables and be able to dance but because of my health I can't. All my personal skills are related to my health and to my 7 herniated discs and spinal cord and to the many migraines that they cause me, that is what prevents me from doing my normal daily life."*[40:23 ¶ 13, DE/GF: Galicia Rural].<br>*"I have physical problems... Due to... Due to the consequences of poliomyelitis, and I would have to do certain physical exercise for muscle maintenance, right? And I've always been... You know, very lazy about exercise. Maybe because you've been there too much... having had a lot of work, you left it until the last minute and you know, and I'm still the same, it takes a lot of effort for me to do physical exercise, but it has to be my purpose. "I can't walk or do standardized gymnastics, because I can't cheat when exercising."[74:38 ¶ GF Aragón Rural].*<br>*"I have not done sports in my life. Because my job and my life circumstances have not allowed me to play sports."[16 ¶ 161 GF: Andalucía Urbano_1].*<br>*"The physical activity that we had is not what we have now: whether we like it or not, there is a lot of sedentary lifestyle, why? Because we have a lot of office jobs, because we work long hours and we don't have time to go for a walk, or... So, the foods are also more processed, they are not healthy"*[85:57 ¶ 366, GF: Catalunya Urbano]<br>*""I would like to walk more and climb hills, but I can't because of my health, I am quite reserved and I would like to be more cheerful and go see my daughter and grandchildren."*[40:24 ¶ 11, DE/GF: Rural Galicia]<br>*"It depends on the volume of work, but that of [regular physical activity] So I do everything wrong. So, if I have to do some of the work, I put one thing first, I put another thing first, and in the end when I realize, I have messed up in the end, so I see work addiction as a difficulty,"*[73:13 ¶ 596, GF: Andalucía Urbano_2]

		"Well, at the moment I'm doing quite well, with a couple of ailments due to a couple of problems with my bones, but well, at the moment I'm working a lot and I have a problem with osteoarthritis, which is bothering me a lot [touches his legs] , there are days when it has me a little [expression of concern] I can hardly work, but hey, we have to go! At the moment we are poor, so we have to fight and keep working." [87:45 ¶ 237, GF: Galicia Urbano]<br>*"Obviously you have to be willing to exercise, but...For us, something as simple as having a dog at home forces me to go for a walk at night."* [87:85 ¶ 589, GF: Galicia Urbano]<br>*"Let's see, I have never been a great athlete, but... Yes, I did my things, I moved, and I notice that a lot now, that the easy answer is: Well, do sports!... Yeah, I don't know, I can't finish find the moment or... the will... no,... no. And well, physically I feel good"* [19:4 ¶ 88, GF: Rural Basin_2]<br>*"My hobby is dancing, but with my life and my illness I can't do it anymore and for many reasons I can't do it anymore. Why can't I do, well, for a few years now, neither sport, nor... walk... nor... or go visit things... why can't I walk, then, well, I feel very limited."* [84:13 ¶ 169, GF: Catalunya Rural_1]<br>*"We are losing something that is natural with age and that is flexibility and elasticity. Of course, when you get that back, you become young again. You come back again, because of course I have the flexibility right now that I did when I was 30 years old. Because those of us who have played sports and have not practiced stretching much, which is one of the things that is practiced a lot in yoga, are getting shorter with our muscles. All of us who have done a more or less intense sport. The boys, who play soccer, I have cycled, I ski, I do everything when I can, because I am not in the habit of stretching my muscles. So with yoga I have regained it, and that has made me feel much younger. Because now I can do things that most people my age can't do. I can jump, I can climb... I can jump a wall, I can do that, because I don't get hurt, because I have a long stride and it doesn't cause an injury or things of that type. So I wanted to er... Point out that within my activities, within how well I feel, I owe a large part of it, apart from sport in general, to the discipline of yoga."* [35:33 ¶ 169, GF: Andalucía Urbano_1]<br>*"We are active! "In the towns, above all, in the towns because you always find something to do."* [6:18 ¶ 337, GF: Castilla y León Rural]
opportunity	**3**<br><br>**Physical Opportunity: Addressing Resource Accessibility Challenges to Maintain Health.**	*"My current state of health is bad. Bad, I'm going through depression [he seems a little down] and that's why I asked him why there are no psychologists in social security. I have been in psychiatric treatment and I ask the psychiatrist. Now they will send me to... a psychologist. And he says no, and I say why? And he says why isn't there. That was the answer."* [16:4 ¶ 121 – 125 GF: Rural Basque Country]<br>*"I take psychotropic drugs and I wouldn't want to take them, I think I might not have to take them if I could afford to go to therapy, I don't know, being able to tell how I feel... however, well... I take medication to alleviate those... emotional ups and downs, it's citalopram. Because they never make an appointment for the psychologist."* [80:83 ¶ 270, GF: Castilla y León Urbano]<br>*"I just had dialysis and I have pain. And then if I have to talk about the public health service... I am very dissatisfied with primary care. Because... It doesn't flow like it did years ago, right? And then, not to mention the emergency service! That's an odyssey. I've had to go to the emergency room five times and... The first time, four hours. The second time, five hours for a doctor to come. And with pain from renal colic, I left there.... That is, not six hours spent in the emergency room, with which I say: I'm going to become unsubmissive! Because you pay taxes and then you don't see benefits, what are we doing here? Very badly. "Emergency service, fatal."* [16:20 ¶ 260 GF: Rural Basque Country]<br>*"Maybe those basic things, like sleeping well, being overweight, which affect other things in your health, should have more direct access in health centers to treat them. Because it would also be a saving. For example, I know a neighbor who had surgery on her knees. And she's really overweight, you know? In the end they are expenses, too, medically. And it affects her, like many other people, indirectly to her health. If they dealt with these types of things in a more accessible and accessible way, perhaps the general situation would improve."* [6:71 ¶ 868 GF: Rural Balearic Islands]<br>*"I have bone disease and bone disease is a rich man's disease. *What I need are massages, I need to lead a very calm pace of life, I need good posture, I need to eat well, the better, for the collagen, and well, mine is: Cling, cling, cling! [pay, pay and pay]"* [85:52 ¶ 326, GF: Catalonia Urbano]<br>*"I think the first... the... a healthy person. Or a health center. He would have to be the family doctor. But I think that's not the case... Why if... if I go to my doctor and tell him: I would like to include some... some variant of some type of diet, what do you advise me? He tells me: "You're not sick, why do you come here with all the people I have? This is clinical" but it should be that way."* [16:20 ¶ 260 GF: Basque Country_Rural]<br>*"I went to rehab in the hospital and had a fatal experience, so I decided not to return. They did, well, they did a differential nerve block and I had a terrible time, I had syncope, I lost my appetite and the doctor was constantly calling me from the hospital... which is a very strange thing... and... just after fifteen days had passed, I He said, do you want us to try some currents and such? And I say: no, you electrocute me."* [7:1 ¶ 354, GF: Castilla y León Rural]<br>*"For example, I am currently with a nutritionist. I want to lose weight. I feel well in all aspects of my life, I feel well physically, but I am aware that I would need... That I would need and I want to lose a little weight. That's why I'm with a nutritionist."* [1:29 ¶ 197 GF: Andalucía Urbano_1]<br>*"If we had fewer options on the candy and food market, we would be healthier and slimmer. If there weren't chocolate donuts and all those cupcakes, and all those... accessible... Would we be thinner, prettier?"* [26:32 ¶ 531,]GF: Rural Catalonia]<br>*"Information one step further would be nice, right? A few more tricks, in addition to the basic information, such as, for example, sugar substitutes, that you have, or that we have in general. And information that is more difficult to access and that in the end is the lack of direct specialists found in the most official fabric. Because you go to the doctor and he's going to give you the same boring diet as always."* [6:28 ¶ 449, GF: Baleares Rural]<br>*"I don't think so because before I always ate out for work and now I eat at home because I love the garden, so I eat much better now than before."* [7:14 ¶ 326, GF: Castilla y León Rural]

		"Sugar has always been consumed without any problem, right? And as children we have had sugar and such. And now it has very bad press. Well, they must have done studies, watching and such. So, it's hard for me to understand, I mean I have a granddaughter, she's going to be one year old and she hasn't tried sugar yet. And I say: oh man, that has no basis, right? Her mother, that is, my daughter, is a health worker, she is an informed person, and she decides that her daughter does not try sugar, does not try I don't know what and I compare it with my... [laughs], because in my childhood, sugar was part of from day to day"[1:29 ¶ 197 GF: Andalucía Urbano_1]<br>*"Food is very important, what happens is that, damn, and I look like the pesetera [expression to indicate that gives a lot of importance to money] but we return... I return to money! Because I know a lot about eating well, I know how to make menus of everything, eat vegan, and my health hasn't changed. But, of course, about 150 euros that I can spend a month eating alone, okay? It costs 374 euros to be able to eat healthy. "Eating healthy is very expensive!"*[185:48 ¶ 314, GF: Catalonia Urbano]
	**4**<br><br>**SOCIAL OPPORTUNITY: Balance in Social and Personal Dynamics: A Reflection on Health and Relationships.**	*"It is essential, you will always be better if you have company, if they support you, you are happier, it is easier to face everything and find a balance. And being out of this balance is a less healthy state. If you do everything that contributes to your personal balance, it is positive."*[6:59 ¶ Rural GF in the Balearic Islands]<br>*"The problem I have are family arguments, at work, and things like that... Ugly... And you make noise with the parents, with my partner because we are arguing every now and then over stupid things and it is a negative sphere and... And look... It makes me look bad with the family, good look bad with everyone. [expresses sadness]"*[82:5 ¶ 169 – 171, GF: Catalunya Rural_1]<br>*"External factors affect me a lot... Obviously my family and my work environment, right? There are people who are toxic, right? There are people who give you bad vibes and your morale is up to date... let's see... let's see if it's positive, you let it go, but there are people... there are people who always hit you in the face. Achilles heel."*[*84:81 ¶ 994 – 998, GF: Catalunya Rural_1*]<br>*"Well, I slept wonderfully until my son was born 21 years ago! Since then I have been a very light sleeper, very, very light. My son coughed a little and my eyes opened and he coughed a second time and he was already there. "It always happened to me."*[1:52 ¶ 612, GF: Andalucía Urbano_1]<br>*"Deny, it is a reality, being overweight, it must be said, is not healthy. And that is what needs to be improved and I am there, but it is difficult for me. Well, it's hard for me. Because I like delicious things, because my activity has changed after having my son, it brought changes in my life."*[6:1 ¶ 217, GF: Baleares Rural]<br>*"Well, I have two children and financially they are my biggest concern and I don't have time to do things for myself, I have to fight for them, because they need someone, you! So, the two children invade me and what I do to improve habits is take them to the park, rest when they have fallen asleep, be mentally very well, very prepared... That is to say... if I have this problem, I have to solve it somehow. way, well I solve it, and there are times...well, I'm telling you, you can't find a moment for yourself."*[73:14 ¶ 598 – 602, GF: Andalucía Urbano_2]<br>*"I have not practically lived with my mother until I was fourteen years old. And now, she is 94 years old and my sister and I are taking care of her for months, right? She was four months old and I was four... of course, my mother is 94 now, so I do notice that I am under tension when I am with her, one... here [touches her chest] when she is with me because, she needs, she wants you to be there for her ... I think they basically need 24 hours of care, that you listen to them 24 hours and of course, well it's [touches head] and I'm bad... that is... Affected. With one pressure we go here [touches his chest], I mean... poof! [she seems moved] and of course, I mean... I've met her now... I mean, I've met her [makes the symbol in quotes with her fingers] yes, I was going to see her, it was once a year, I mean... but no... It's just that I haven't lived with her before... so now... it's very hard. For me it's super hard. And I do understand that they need love, and my mother is also used to being alone."*[16:55 ¶ 1149– 1160, GF: Rural Basque Country]<br>*"I like to be always out of my house... but... my wife can't walk as much as she should, due to physical problems, and so if I leave, she would be left alone at home and well, in order not to leave her alone, I I stay at home, I don't do anything, and I say: come on, come on, we'll go later, we'll go out later, the pain will go away, but no."*[3:25 ¶ 412, Aragón Urbano]<br>*"For me, having good relationships is positive for my health, of course. That I can, in certain cases, comment to my partner, for example, hey, my head hurts... an example, and not tell me: "oh, you're starting to... ", well not: "it hurts today... and that?... Have you had a bad day? For example, call the doctor in certain cases... who can help you with something, I can't give you anything. So the bond with someone is going to be positive, it is going to be the first contact... be it my mother... be it my partner... be it my daughter... whoever surrounds me. In short, understand!eithern! I see an understanding, in life there are moments of great affection and others of more sadness it seems."*[17:52 ¶ 989, 155-242, GF: Urban Basque Country]<br>*"The couple. It's what brings me happiness. Being well with my partner is important to me... which doesn't mean that I wouldn't be happy if I didn't have her... but for me, if I have her it brings me happiness."*[73:57 ¶ 1678, GF: Andalucía Urbano_2]<br>*"For me, couple life influences... both physical and mental well-being."*[75:124 ¶ 741, GF: Baleares Urbano]<br>*"After a pregnancy, maybe our body is no longer the same, the hormones, this, the other as it happens... that if it leads us to other diseases, with pressure, arteries, etcetera, etcetera. And when I was younger I did a lot of sports and I have realized that since I am a mother and I move less, because I mean, I don't do... I do sports like running after the children and doing something else that I do out there. ."*[19:8 ¶ 104, GF: Rural Basin_2]<br>*"Well, I also have a teenage son, and I can't change that stress anymore...! There is no remedy for that."*[87:74 ¶ 452, GF: Galicia Urbano]<br>*"Our life is directed from the beginning and through cinema, television, all the stories that they put in us, they create a way of living that we would not have to do. In other words, we are under stress, the children have to leave school, we have to add four activities, they have to do I don't know what, we have to take them to I don't know what... and that, when we can no longer make it compatible with our work schedule. , time with our wife. That leads to stress in the end, which often forces us to eat that junk food, that fast food. We don't have time to prepare everything you need.*

		And in the end, when the children are finished and gone, if they are lucky enough to have good jobs and have total independence, then we parents can begin to rethink a little about what we want to do with our lives, but before that, we are on our own. children."[73:16 ¶ 606, Andalucía Urbano_2]<br>*"I'm not into social media nor do I follow anyone, really... but... well... tik tok, which my daughter teaches me and little else, and... what I know, I listen to people, I read... I watch TV, of course , I watch a lot of movies, a lot of series, ... and of course everything influences you, your mother influences you, your friends influence you, your environment influences you... but you, you have the mind to let it not be that way, and I believe that you have to know what is important to you. And that's it."*[185:48 ¶ 314, GF: Andalucía_Urbano_2]<br>*"Well, I think we have a problem with happiness here. Happiness has become so fashionable... Happiness has been so overrated that it is like you have an obligation to be happy. And you also have to accept when you are sad, I think the social network has done a lot of damage. And the social network is basically magical reality and magical reality that is not real."*[85:91 ¶ 677, GF: Catalunya Urbano]<br>*"Because of my reserved and introverted nature, I have little social life and that is why I close myself off from people and it is difficult for me to have a social life and meet people, and normally if someone gets angry and starts arguing with me, I just move on from the topic and don't give it importance. ."*[36:37 ¶ FROM: Catalunya Rural_1]<br>*"There are people who are not able to say: I am not going to get out of this. Need extra help. The brain is an organ and when it breaks down, it breaks down just like the others."*[84:38 ¶ 303, GF Catalunya Rural_1]<br>*"In the sociable, as some people who are doing sports say...I can't do any sports... eitherbe...what is zero...[expresses frustrationeitherny sadness throughout his speech] a few years agoñok, neither sport nor...walk... or... or go visit things... because I can't walk, so, well, I feel very limited... and... well, I lost the job I had, I lost it because of my illness... because I'm from operation to operation to operation and I I'm not old enough for so many operations. So I the htothing I have is being at home watching TV and social things, well with the family, and that's it.to. And that is my life and nothing moretoyes.Zero, life, zero, what I have at home and little else. Yes, I already enjoy it, in my own way and then the society that helps you a little bit. "*[18:06 ¶ 155, GF: Catalunya Rural_1]
Motivation	**5**<br><br>**REFLECTIVE MOTIVATION: Adaptation to Illness and Conscious and Planned Personal Care.**	*"I am learning a lot about integrative medicine, because I find it super interesting, in many aspects and... I think at a given moment it can help a lot... And I also really like the field of nutrition, as a healthcare professional I really like to find out how How the body works, our hormones, how they work, all that, and well, I think I've reached a point in my life that... before I didn't think about studying, that is, studying, just like I'm doing. now."*[16:19 ¶ 252 GF: Rural Basque Country]<br>*"For example, for me, the most essential thing is that... And you see that all my children who do not argue... do not have problems... well, in principle I have been lucky because now they get along very well and get along with me like a friend, because... I That's what I have to say: I'm a very good friend... no... I'm not the typical one... who calls me for anything."*[7:33 ¶ Rural GF in Castilla y León].<br>*"I come and go all day, I mean, scales above, scales below. And sometimes, well, I like to rest after sunset. There are days when you end up crunched, I mean... You have to rest! Sometimes instead of half an hour, there are four hours, which I have done, of course, this is not good either. On the couch for four hours, and of course, four hours I don't know... I don't need to rest so much"*[6:49 ¶ 632, GF: Baleares Rural]<br>*"Me personally, physically, well yes, I am in a group, I do mountaineering... Well, mountains, not mountaineering of eight thousand meters, no. I go, but I walk... Suddenly I got into a patchwork course. At first I said, oh, what nonsense... Well, no! It has helped me a lot, developing my imagination more, occupying my time, and also recreating myself in it, right? Apart from one personally, one does have family and personal problems, but not focusing, but rather having other additional activities. It could be that, physical, mental, everything, I think that is very important, having activities."*[17:24 ¶ 398 in Basque_Urban Country]<br>*"I went through a bad time due to some abortions and no one around me could help me, because they saw me and I was like: "no, I'm fine, nothing happens." I went to a meeting with friends, some friends invited me, I went to a collective meditation where we talked about the moon, the rays that enter your body, and come on... something like that was very silly... I felt super ridiculous and I I was sitting and thinking: "My goodness... what nonsense... What am I doing here?" but then, like I managed to get inside myself, think, that is... they helped me a little to get all that emotion out, I started crying like a crazy person, they took out all the emotion, all the, everything that I had inside , of sadness... I had like a farewell to those babies that were inside me. I said goodbye to them. And two months later I got pregnant. Maybe it had nothing to do with it, or maybe it did. But emotionally for me, it was a very big release."*[19:41 ¶ 217, GF: Rural Basin_2]<br>*"My own demands and daily dynamics allow me to carry out sports activities, achieve my purposes and my goals, such as doing a small marathon, a bicycle race, and this affects me in having good health and good mental activity."*[36:25 ¶ 160 FROM: Rural Catalonia_1]
	**6**<br><br>**AUTOMATIC MOTIVATION. Instinctive Impulses and Habits: Personal Aptitudes and Desires for Individual Balance.**	"Yes, my health depends a little on the attitude, the attitude of each person, which is a personal issue; the attitude depends on us [6:36 ¶ GF Rural in the Balearic Islands].<br><br>*Sometimes I do things that I don't feel like doing or that I don't feel comfortable with because it's hard for me to say no. [D. Written¶ GF Rural in Galicia].*<br><br>*The most important thing I have is the support of all my people around me. And what I receive from them. For me, that is very important, it is... being there for each other, important. And without it I am not able to live."*[1:4 ¶ 118 GF: Andalucia_Urbano_1_Triang].

		"So I think that due to personality... I consider myself a sponge, when it comes to... Well, when I receive news, or when I receive [?] in general, well I try to absorb everything. "It's one of the problems I have." (17:13 ¶ Urban GF in the Basque Country).<br>*"Relationships bring positive things to me, like sports. In other words, since I also like to play sports in a group, I need to interact, get people together to play sports, go out to have a beer, with my neighbors, and all of that balances me a lot, for me human relationships are fundamental. , in a good level of health."* (15:57 ¶ 650 GF: Andalucía Urbano_1)<br>*"Being sociable and alternative is a plus for practicing sports with different people of different ages and genders."* (2:90 ¶ 918, GF: Catalunya Rural_2)<br>*"Yes, [covid-19] the pandemic has greatly affected all of us, but well for most of us, if not all of us. And that... many things are missed in terms of meeting people, and friends, you see them less, you go out less frequently, which also lowers the pace of doing sports, of being able to walk more.* (13:10 ¶ 216, GF: Rural Basin)<br>*"Of course they take care of her, it is important that they take care of us, my mother, and to feel loved and it is very important."* (2:77 ¶ 793, GF: Catalunya Rural_2).<br>*"I'm going to make a case that it motivated me to do yoga. Because I had heard about that, but I thought that was nonsense. That was going out and stretching my legs a little... But they once invited me to a class, and a woman who was very old, she was very old, stood next to me and I thought to myself, how is that lady going to do yoga? Well, the lady did the yoga perfectly. When I finished, I had the indiscretion or the audacity to ask her how old she was. And the woman tells me: I am eighty-three years old. And I... Then I went cold. 83 years old, really, ma'am? I mean, the lady knows that today she is, I think, 86 or 87 and she continues doing yoga. From my point of view, seeing that lady was a clear example for me. So that's why I turned to yoga, which has given me a lot."* (1:35 ¶ 225 in Andalucia_Urbano_1_Triang).<br>*"My way of being social has made me do more group sports and meet friends who have given me pleasant and positive moments."* (D. Written ¶ GF Urbano in Andalusia).<br>*"I am controlling in all daily situations, I want to control everything and sometimes it causes me uncomfortable situations with other people and nervousness. I use sport because it helps me disconnect and relax, to disconnect with my controller."* (44:19 ¶ FROM GF: Baleares Rural_ 2).
